# PROTOCOL: Global elder abuse: A mega‐map of systematic reviews on prevalence, consequences, risk and protective factors and interventions

**DOI:** 10.1002/cl2.1227

**Published:** 2022-04-27

**Authors:** Christopher Mikton, Marie Beaulieu, Yongjie Yon, Julien Cadieux Genesse, Kevin St‐Martin, Mark Byrne, Amanda Phelan, Jennifer Storey, Michaela Rogers, Fiona Campbell, Parveen Ali, David Burnes, Tova Band‐Winterstein, Bridget Penhale, Mark Lachs, Karl Pillemer, Lilly Estenson, Kelly Marnfeldt, Jessica Eustace‐Cook, Anthea Sutton, Francis Lacasse

**Affiliations:** ^1^ Department of Social Determinants of Health World Health Organization Geneva Switzerland; ^2^ École de travail social, Faculté des lettres et sciences humaines Université de Sherbrooke Sherbrooke Québec Canada; ^3^ Research Chair on Mistreatment of Older Adults Sherbrooke Québec Canada; ^4^ World Health Organization Regional Office for Europe Copenhagen Denmark; ^5^ School of Nursing and Midwifery, Trinity College Dublin Dublin Ireland; ^6^ School of Psychology University of Kent Canterbury Kent UK; ^7^ Department of Sociological Studies University of Sheffield Sheffield UK; ^8^ School of Health and Related Research The University of Sheffield Sheffield UK; ^9^ Rotman Research Institute, Factor‐Inwentash Faculty of Social Work Affiliate Scientist, Baycrest University of Toronto Ontario Toronto Canada; ^10^ Department of Gerontology Faculty of Social Welfare and Health Sciences University of Haifa Haifa Israel; ^11^ School of Health Sciences University of East Anglia Norwich UK; ^12^ Geriatric Medicine Weill Cornell Medicine New York New York USA; ^13^ Cornell Institute for Translational Research on Aging Cornell University New York New York USA; ^14^ Leonard Davis School of Gerontology University of Southern California Los Angeles California USA; ^15^ Library, Trinity College Dublin Dublin Ireland; ^16^ School of Health and Related Research University of Sheffield Sheffield UK; ^17^ Hôtel‐Dieu de Sherbrooke Sherbrooke Québec Canada

## Abstract

This is the protocol for a Campbell systematic review. The objectives are as follows: to produce a mega‐map which identifies, maps and provides a visual interactive display, based on systematic reviews on all the main aspects of elder abuse in both the community and in institutions, such as residential and long‐term care institutions.

## 1 BACKGROUND

### 1.1 The problem, condition or issue

The World Health Organization defines elder abuse as a single or repeated act or lack of appropriate action, occurring within any relationship where there is an expectation of trust which causes harm or distress to an older person (WHO, 2021a). The main forms of elder abuse generally recognized and which can occur in the community and institutional settings are physical, psychological, sexual, financial/material and systemic/organizational abuse and neglect, as well as poly‐victimization.

Around one in six people 60 years and older experience some form of abuse in community settings annually. Some 12% suffer psychological abuse; 7%, financial abuse; 4%, neglect; 3%, physical abuse; and 1% experience sexual abuse (Yon et al., 2017).

Rates of elder abuse are higher still in nursing homes and other long‐term care facilities, with two in three staff reporting that they have committed abuse in the past year—33% psychological abuse, 12% neglect, 9% physical abuse and less than 1% sexual abuse. Most of these data, however, come from high‐income countries (Yon et al., 2019).

It is predicted that by the year 2050, the global population of people aged 60 years and older will more than double, from 900 million in 2015 to about 2 billion, with most older people living in low‐ and middle‐income countries. If the proportion of elder abuse victims remains constant, the number of victims of elder abuse will increase rapidly (WHO, 2021a).

A recent systematic review and meta‐analysis of elder abuse in community settings found no significant difference in the overall prevalence between older women and older men globally (Yon et al., 2017). However, only 32 of the 52 studies included provided gender breakdowns and most were from high‐income countries.

An analysis of studies that included elder abuse prevalence rates of women in the community found that globally past year prevalence of overall abuse was 14.1%; 12% for psychological abuse; 4% for neglect; 4% for financial abuse and 2% for both sexual abuse and physical abuse. In relation to women, the abuse is often a continuation of intimate partner violence into old age (ʻintimate partner violence grown old’) (Lundy & Grossman, 2009). While some studies have indicated that abuse decreases with age, others have found that abuse continues at much the same rates in older age (Catalano, 2012; Wilke & Vinton, 2005; Zink et al., 2003).

Elder abuse can result in serious consequences including physical injuries (Lachs & Pillemer, 2004; Mouton & Espino, 1999), emotional and psychological distress and mental health problems (Comijs et al., 1999; Weeks & LeBlanc, 2011), decline in cognitive functioning (Dong et al., 2014), placement in nursing homes, financial devastation, as well as the loss of family solidarity and trust (Pillemer et al., 2015). Elder abuse affects not only the victims themselves but also their family and larger society. These negative health, economic and social outcomes can further exacerbate existing illness leading to the increased risk for institutionalization, hospitalization, morbidity and mortality (Baker, 2007; Baker et al., 2009; Dong & Simon, 2013; Dong et al., 2009; Lachs et al., 1998; Pillemer et al., 2015; Schofield et al., 2013).

Some of the risk and protective factors supported by the strongest evidence are at the level of the individual (Pillemer et al., 2016; Storey, 2020). In relation to the characteristics of victims, they include:

- Risk factors: functional dependence/disability, poor physical health, cognitive impairment, poor mental health and low income/socioeconomic status;
- Protective factor: social support/social embeddedness.

In relation to the characteristics of perpetrators, they include:

- Risk factors: mental illness, substance abuse and abuser dependency on victim (e.g., financial dependency, dependency for housing).

The evidence supporting risk and protective factors at the level of relationships, the community and society is generally weaker. These may include type of relationship (e.g., spouse partner, children and children‐in‐law), but these vary by type of abuse and by culture; marital status (though findings are mixed); and levels of ageism in society. The evidence for many other risk factors is either weaker or mixed—for example, gender of victims, age, race/ethnicity (Johannesen & LoGiudice, 2013; Pillemer et al., 2016; Storey, 2020).

Many different types of interventions to prevent, detect and respond to elder abuse have been implemented. These include, for instance, public and professional awareness campaigns, school‐based intergenerational programmes, caregiver support interventions, residential care policies to define and improve standards of care, caregiver training on dementia, mandatory reporting of abuse to authorities, and psychological programmes for abusers. Few of these interventions, however, have been shown to be effective in high‐quality studies (Ayalon et al., 2016; Baker et al., 2017; Fearing et al., 2017; Pillemer et al., 2016; Ploeg et al., 2009).

Based on lower quality studies, a 2016 review singled out five types of interventions as being promising: (1) helplines, the most widely used interventions in most countries; (2) caregiver interventions, which provide services to relieve the burden of caregiving; (3) multidisciplinary teams which coordinate care and reduce fragmentation in response to elder abuse; (4) money management programmes which aim to reduce risk of financial exploitation, especially of people with cognitive impairment; and (5) emergency shelters (Pillemer et al., 2016).

Although the prevalence of elder abuse is high and its consequences serious, it is widely recognized that the global political priority of elder is not commensurate with the burden of the problem globally (Mikton et al., 2017; Phelan, 2020; Roberto, 2016). The World Health Organization, as part of the United Nations Decade of Healthy Ageing (WHO, 2021b), has decided to step up its activities to address elder abuse, increase the resources devoted to it, and develop a strategy to address it.

### 1.2 Why it is important to develop the mega‐map

Although major gaps in research and data exist—for instance regarding the nature and prevalence of elder abuse in lower income countries and high‐quality evaluations of existing interventions, the number of publications on elder abuse has been growing, with a 36% increase in the number of publications in the decade 2008–2017 over 1998–2007 (Sweileh, 2020). However, the global evidence on the prevalence, consequences, determinants and interventions of elder abuse is often scattered and fragmentary, difficult to locate and unusable by policy makers and practitioners (Fearing et al., 2017; Storey, 2020; Yon et al., 2017). To date, no attempt has been made to map the literature on elder abuse. A mega‐map that provides a visual and interactive display of evidence synthesis studies on the prevalence and consequences of, and risk and protective factors and interventions for, elder abuse in both the community and in institutional settings will serve the following important purposes:

- Increase the discoverability and use of evidence on elder abuse by policy and decision makers, programme commissioners and practitioners in countries;
- Identify areas where more research is needed and guide the commissioning of research in a more coordinated and strategic way;
- Contribute to building an ʻevidence architecture’ or ʻeco‐system’ for the field of elder abuse (Shepherd, 2020; White, 2019);
- Help raise awareness of the problem with a view to increasing its global priority; and
- Help WHO formulate its global strategy to address elder abuse globally within the UN Decade of Healthy Ageing (WHO, 2021b).

### 1.3 The scope of the mega‐map

The scope of the map will cover the following:

1. Prevalence of elder abuse in people aged 60 years and over, organized by type of settings (community or institutional) and type of abuse whose prevalence is being measured (physical, psychological, sexual, financial/material and systemic/organizational abuse and neglect, as well as poly‐victimization);
2. Consequences, organized by type of consequence (e.g., physical health symptoms, psychological/mental health symptoms, social service use and social and economic consequences) and by type of abuse;
3. Risk and protective factors, organized by type of risk or protective factor at the individual victim, individual perpetrator, relationship, community and societal levels, as well as the level of institutions and type of abuse; and
4. Interventions, organized by type of intervention (prevention, detection and response) and type of abuse.

The definition of the scope of, as well as the framework for (see 3.3), the map has benefited from the input of the advisory and stakeholders groups (see Supporting Information Appendix 7 for details on the advisory and stakeholders groups and a report on the stakeholders meeting).

### 1.4 Conceptual framework for the mega‐map

**Figure 1:**
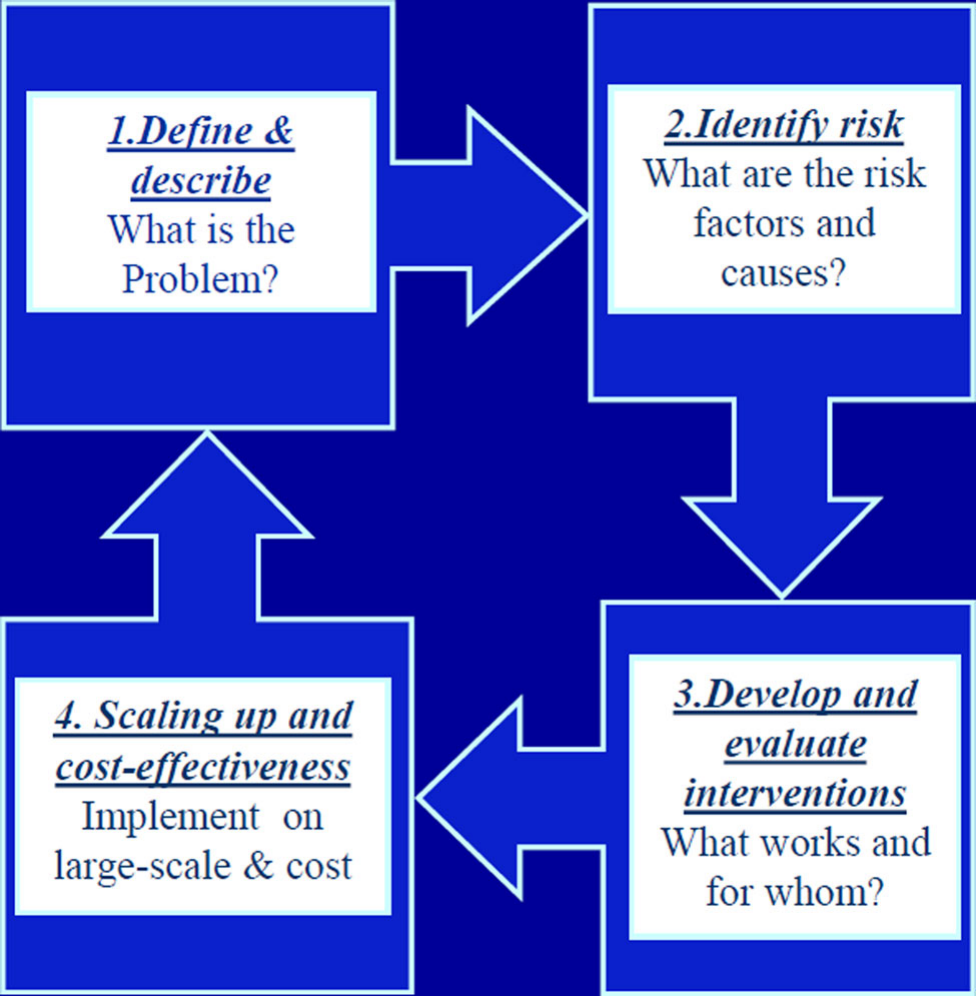
The four steps of the public health approach

**Figure 2:**
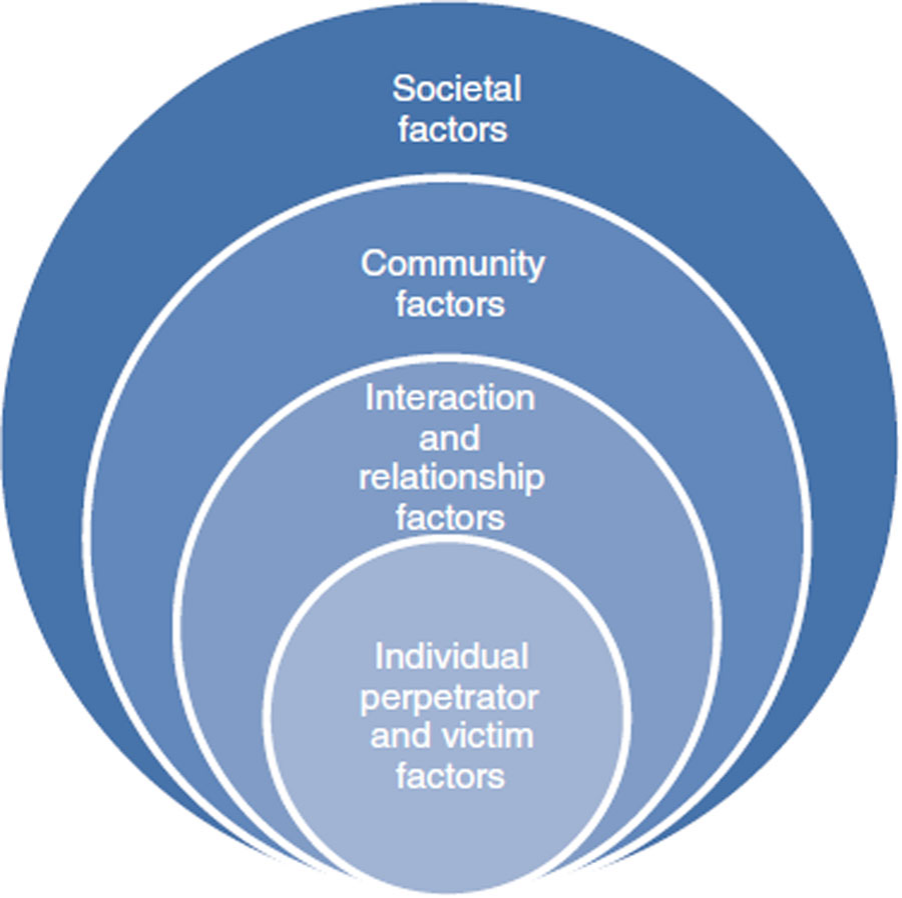
Risk and protective factors organized according to the socio‐ecological model (Labrum & Solomon, 2015)

**Figure 3:**
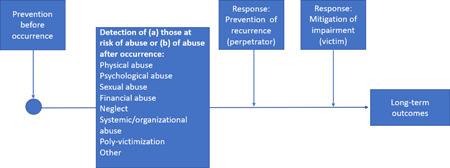
Framework for interventions to prevent, detect and respond to elder abuse

This mega‐map will be informed by the public health approach to the prevention of health conditions, including violence, and will draw on three interconnected aspects of the public health approach: (1) the four steps of the public health approach (Figure 1); (2) the socio‐ecological model, which expands on Step 2 of the public health approach on risk and protective factors (Figure 2); and (3) the distinction between preventing, detecting and responding to elder abuse, which expands on Step 3 of the public health approach on interventions (Figure 3) (Krug et al., 2002; Mercy et al., 1993; Satcher & Higginbotham, 2008).

**Figure 4:**
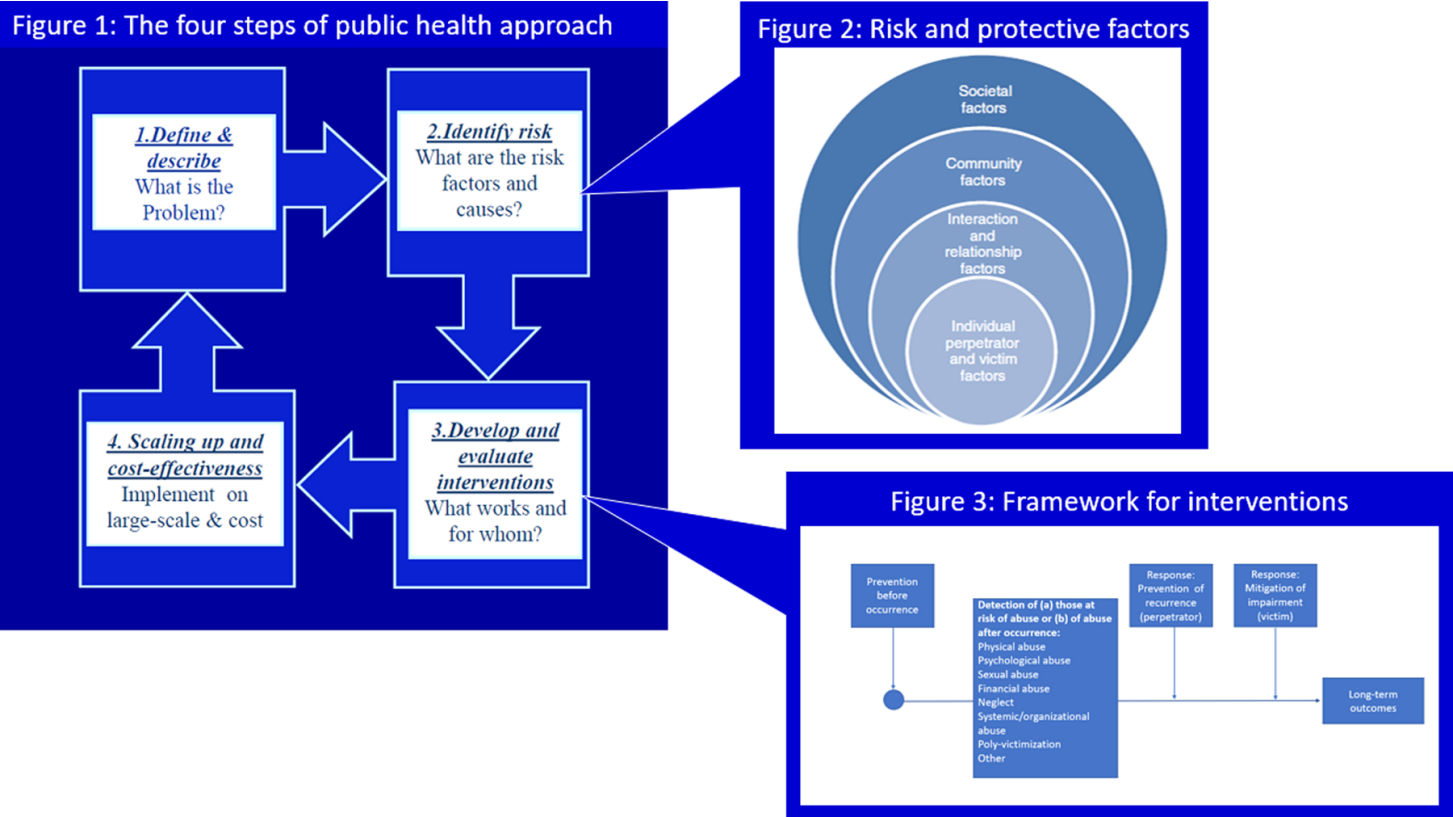
How Figures 1, 2, 3 are connected

*The four steps of the public health approach:* The public health approach is characterized by four key steps (Figure 1):

1. Defining and measuring the problem: uncovering as much basic knowledge as possible about all aspects of elder abuse—through systematically collecting data on the prevalence, distribution and consequences of elder abuse.
  Components 1 and 2 of the mega‐map will address this first step.
2. Determining the causes or risk factors for the problem: investigating why elder abuse occurs—that is, conducting research to determine the causes and correlates of elder abuse; the factors that increase, decrease, or buffer the risk for elder abuse; and the factors that might be modifiable through interventions.

Figure 2 on the socio‐ecological model expands on Step 2 of the public health approach providing a model for organizing risk and protective factors.

Component 3 of the mega‐map on risk and protective factors will address this step of the public health approach.

3. Determining how to prevent, detect and respond to elder abuse by designing, implementing, monitoring and evaluating interventions.

Figure 3—on the framework for interventions expands on Step 3 of the public health approach, providing a framework for interventions.

Component 4 of the map on interventions will address the third—and partly the fourth—step of the public health approach.

4. Implementing, in a range of settings, interventions that appear promising, widely disseminating information and determining the cost‐effectiveness of programmes.

Figure 4 shows how these three figures are connected.

Each of these steps of the public health approach are linked and build on each other to an extent. For instance, if definitions of elder abuse (Step 1) are not clear, elder abuse cannot be measured accurately and its prevalence and distribution (Step 1) cannot be established with confidence. If elder abuse cannot be measured accurately, it will be more difficult to identify risk and protective factors and underlying causes correctly (Step 2). If risk and protective factors and underlying causes are not identified correctly (Step 2), interventions to prevent elder abuse (Step 3) are unlikely to be effective and difficult to implement and scale up (Step 4).

*The socio‐ecological model:* The public health approach draws on the socio‐ecological systems model which helps to identify and explain the multidimensional aspects of elder abuse (Bronfenbrenner, 1979), in particular to help understand and organize risk and protective factors and interventions (Figure 2).

The socio‐ecological approach proposes that human beings are embedded in nested systems related to context and progressively adapt to accommodate to their environment over time. Individuals are affected by, and in turn affect their environments. In addition, reciprocal causation is present, which means individual behaviour moulds, and is moulded by, environment (Phelan & Kirwan, 2020). Thus, the socio‐ecological model allows an integration of individual and environmental factors to enable an examination of risk and protective factors and interventions within complex systems.

Thus, a complex range of factors—at the individual (perpetrator and victim), relationship, community and societal or systems levels—are considered to put people at risk of elder abuse (Figure 2). Identifying risk factors at these four nested and interacting levels helps to make sense of the many interventions and strategies which target these risk factors to reduce elder abuse (Bronfenbrenner, 1992; Krug et al., 2002; O'Brien et al., 2016; Schiamberg & Gans, 2000).

*Classification of interventions:* In addition, we will draw on the distinction between preventing, detecting and responding, which is widely used in public health, to organize interventions as follows (see Figure 3) (Krug et al., 2002; MacMillan et al., 2009):

Interventions that aim to *prevent* elder abuse before it occurs;

Interventions that aim to *detect* people at risk of abuse before it occurs or who have experienced abuse;

This interventions that aim to *respond* to elder abuse after it has occurred, which either seek:

To prevent the recurrence of elder abuse (aimed at perpetrators);

To mitigate the impairment caused by elder abuse (aimed at older people who have experienced abuse).

### 1.5 Existing mega‐maps, evidence and gap maps (EGMs) and/or relevant systematic reviews

No completed EGM on any aspect of elder abuse were identified. There are, however, many relevant systematic reviews on the prevalence and consequences of, and risk and protective factors and interventions for elder abuse. The database maintained by Sherbrooke University in Canada has, for instance, identified 64 systematic reviews covering various aspects of elder abuse or of related issues (https://maltraitancedesaines.com/en/). Our searches will not be in any way limited to this database and will extend across multiple databases. Examples of systematic reviews include the following:

Prevalence:

- Abdi, A., Tarjoman, A., & Borji, M. (2019). Prevalence of elder abuse in Iran: A systematic review and meta‐analysis. *Asian Journal of Psychiatry*, *39*, 120–127. doi:10.1016/j.ajp.2018.12.005
- Arab‐Zozani, M., Mostafazadeh, N., Arab‐Zozani, Z., Ghoddoosi‐Nejad, D., Hassanipour, S., & Soares, J. J. F. (2018). The prevalence of elder abuse and neglect in Iran: A systematic review and meta‐analysis. *Journal of Elder Abuse & Neglect*, *30*(5), 408–423. doi:10.1080/08946566.2018.1523765
- Cooper, C., Selwood, A., & Livingston, G. (2008). The prevalence of elder abuse and neglect: A systematic review. *Age Ageing, 37*(2), 151–160. doi:10.1093/ageing/afm194
- Ho, C. S., Wong, S.‐Y., Chiu, M. M., & Ho, R. (2017). Global prevalence of elder abuse: A metaanalysis and meta‐regression. *East Asian Archives of Psychiatry, 27*(2), 43.
- Sooryanarayana, R., Choo, W. Y., & Hairi, N. N. (2013). A review on the prevalence and measurement of elder abuse in the community. *Trauma Violence Abuse, 14*(4), 316–325. doi:10.1177/1524838013495963
- Yan, E., Chan, K. L., & Tiwari, A. (2015). A systematic review of prevalence and risk factors for elder abuse in Asia. *Trauma Violence & Abuse, 16*(2), 199–219. doi:10.1177/1524838014555033
- Yon, Y., Mikton, C., Gassoumis, Z. D., & Wilber, K. H. (2019). The prevalence of self‐reported elder abuse among older women in community settings: a systematic review and meta‐analysis. *Trauma, Violence, & Abuse, 20*(2), 245–259.
- Yon, Y., Mikton, C. R., Gassoumis, Z. D., & Wilber, K. H. (2017). Elder abuse prevalence in community settings: A systematic review and meta‐analysis. *The Lancet Global Health, 5*(2), e147–e156.
- Yon, Y., Ramiro‐Gonzalez, M., Mikton, C. R., Huber, M., & Sethi, D. (2019). The prevalence of elder abuse in institutional settings: a systematic review and meta‐analysis. *European Public Health Association*, 29(1), 58‐67. doi:10.1093/eurpub/cky093

Consequences

- McGarry, J., Simpson, C., & Hinchliff‐Smith, K. (2011). The impact of domestic abuse for older women: A review of the literature. *Health & Social Care in the Community*, 19(1), 3–14. doi:10.1111/j.1365‐2524.2010.00964.x
- Yunus, R. M., Hairi, N. N., & Choo, W. Y. (2019). Consequences of Elder Abuse and Neglect: A Systematic Review of Observational Studies. Same question as above. *Trauma, Violence & Abuse, 20*(2), 197–213. doi:10.1177/1524838017692798

Risk and protective factors

- Johannesen, M., & LoGiudice, D. (2013). Elder abuse: A systematic review of risk factors in community‐dwelling elders. *Age Ageing, 42*(3), 292–298. doi:10.1093/ageing/afs195
- Pillemer, K., Burnes, D., Riffin, C., & Lachs, M. S. (2016). Elder abuse: Global situation, risk factors, and prevention strategies. *Gerontologist, 56*(Suppl_2), S194–S205. doi:10.1093/geront/gnw004
- Storey, J. E. (2020). Risk factors for elder abuse and neglect: A review of the literature. *Aggression and Violent Behavior, 50*, 101339.

Interventions:

- Ayalon, L., Lev, S., Green, O., & Nevo, U. (2016). A systematic review and meta‐analysis of interventions designed to prevent or stop elder maltreatment. *Age and Ageing*, *45*(2), 216–227.
- Baker, P. R., Francis, D. P., Hairi, N. N., Othman, S., & Choo, W. Y. (2016). Interventions for preventing abuse in the elderly. *Cochrane Database of Systematic Reviews* (8).
- Daly, J. M., Merchant, M. L., & Jogerst, G. J. (2011). Elder abuse research: A systematic review. *Journal of Elder Abuse & Neglect*, *23*(4), 348–365.
- Fearing, G., Sheppard, C. L., McDonald, L., Beaulieu, M., & Hitzig, S. L. (2017). A systematic review on community‐based interventions for elder abuse and neglect. *Journal of elder abuse & neglect*, *29*(2–3), 102–133.
- Mileski, M., Lee, K., Bourquard, C., Cavazos, B., Dusek, K., Kimbrough, K., & McClay, R. (2019). Preventing the abuse of residents with dementia or Alzheimer's disease in the long‐term care setting: A systematic review. Clinical interventions in aging, *14*, 1797. Ploeg, J., Fear, J., Hutchison, B., MacMillan, H., & Bolan, G. (2009). A systematic review of interventions for elder abuse. *Journal of Elder Abuse & Neglect*, *21*(3), 187–210.
- Mohd Mydin, F. H., Yuen, C. W., & Othman, S. (2019). The effectiveness of educational intervention in improving primary health‐care service providers' knowledge, identification, and management of elder abuse and neglect: A systematic review. *Trauma, Violence, & Abuse*, 1524838019889359. Rosen, T., Elman, A., Dion, S., Delgado, D., Demetres, M., Breckman, R., & National Collaboratory to Address Elder Mistreatment Project Team. (2019). Review of programs to combat elder mistreatment: focus on hospitals and level of resources needed. *Journal of the American Geriatrics Society*, *67*(6), 1286–1294.

An EGM on interventions for elder abuse is currently being developed by a team at Lanzhou University in China including Jieyun Li, Liping Guo, Howard White, Jingwen Li, XiuxiaLi, and Kehu Yang. We have been collaborating with them very closely and are striving to align the component of our mega‐map that addresses interventions with theirs to ensure that they are both compatible and complementary. Moreover, we have been reviewing each other's Title Registration Forms and Protocols and the Lanzhou team will help us with our searches in Mandarin.

Although our mega‐map will complement Lanzhou's team EGM on interventions for elder abuse, it will also differ from theirs in the following respects: (a) our mega‐map covers the prevalence and consequences of and risk and protective factors for elder abuse, in addition to interventions; and (b) will be based only on systematic reviews. The Lanzhou team's EGM on interventions will include information on primary studies and will complement this mega‐map by providing a more fine‐grained mapping of interventions and outcomes measures.

## 2 OBJECTIVES

The overall aim is to produce a mega‐map which identifies, maps and provides a visual interactive display, based on systematic reviews on all the main aspects of elder abuse in both the community and in institutions, such as residential and long‐term care institutions.

The specific objectives are to:

1. Identify, appraise and map available systematic reviews on the:
  – Prevalence of elder abuse;
  – Consequences of elder abuse;
  – Risk and protective factors for elder abuse at the levels of the victim, perpetrator, relationships, community and societal levels; and
  – Interventions to prevent, detect and respond to elder abuse;

This will done in both community and institutional settings and an overview will be provided in a summary report;

2. Develop a taxonomy of interventions to prevent, detect and respond to elder abuse and outcomes of such interventions.
3. Provide information in the mega‐map which summarize the aim of the systematic reviews included, the methods used, its main findings and conclusions, an appraisal of the quality of the review, and a link to it.
4. Develop two visual and interactive maps based on the systematic reviews: The first will consist of a matrix with rows and columns with systematic reviews in the cells; the second will consist of map of the world with the distribution by countries of the primary studies included in the systematic reviews.

## 3 METHODS

### 3.1 Defining mega‐maps and EGMs

EGMs are maps of a specific sector or sub‐sector which typically includes both systematic reviews and primary studies. Mega‐maps are maps which are broader in scope covering a large sector or several sectors that includes only systematic reviews and other maps. Produced using the same systematic approach as systematic reviews, both EGMs and mega‐maps usually show what evidence is there, not what the evidence says (White et al., 2020).

The mega‐map on elder abuse will include systematic reviews; no other maps have been identified.

### 3.2 Population

Older people, defined as people 60 years and older, living in both the community and in institutional care settings, will be the main population of interest. Institutional care settings refer to institutions in which long‐term care is provided; these may include community centres, assisted living facilities, nursing homes, hospitals and other health facilities; institutional care settings are not defined only by their size (WHO, 2015).

Filters in the map will allow studies which focus on sub‐groups of the main population of interest to be selected. These sub‐groups will be defined, for instance, by sex, disability (including cognitive impairment), setting (community or institution), WHO‐defined regions of the world, and World Bank‐defined country income level).

### 3.3 Framework for the mega‐map

The framework for the map has been developed by drawing on typologies in existing systematic reviews and the expertise of the team working on the map, several members of which are world‐renowned experts on elder abuse. The development of the framework has also been informed by a meeting of the advisory and stakeholders group which reviewed an earlier version of the framework and helped define the scope of the mega‐map (see Supporting Information Appendix 7). In addition, this framework has been revised more than once after testing it using a sample of systematic reviews. See Supporting Information Appendix 1 for definitions and examples of the terms used in the framework.

#### 3.3.1 Rows in the framework

Given that this map focuses on prevalence, consequences and risk and protective factors, in addition to interventions, the rows will consist of more than just interventions. Hence, we do not refer to the rows as being ʻinterventions’ as is customary practice with EGMs on interventions only, but as ʻrows’.

The rows will include the following:

- Studies on the prevalence of elder abuse divided into studies on the prevalence in:
  ∘ The community; and
  ∘ Institutions;

- Studies on the consequence of elder abuse divided into studies on:
  ∘ Service use (e.g., emergency departments, hospitalization, etc.);
  ∘ Mortality;
  ∘ Physical health symptoms;
  ∘ Psychological/mental health symptoms;
  ∘ Social and economic consequences (e.g., placement in institution, social isolation and loneliness, loss of economic resources, etc.).
  ∘ Other consequences of elder abuse.

- Studies on risk and protective factors for elder abuse, divided into studies on:
  ∘ Individual level factors related to the victim (e.g., age group, sex, dependency, etc.);
  ∘ Individual level factors related to the perpetrator (age group, sex, drug and alcohol dependence);
  ∘ Relationship level factors (e.g., poor or conflictual relationship in family/outside family, financial dependence on older person);
  ∘ Community and societal level risk factors (e.g., discrimination, neighbourhood violence);
  ∘ The characteristics of care institutions (e.g., institutional tolerance of aggression, poor or inadequate training of staff, etc.).

- Studies on interventions to prevent, detect and respond to elder abuse, each divided into the following, based on the target of the intervention:
  ∘ Older people;
  ∘ Professional caregivers;
  ∘ Non‐professional caregivers (e.g., family, friends, etc.);
  ∘ Perpetrators of elder abuse (for response only);
  ∘ The general population (for prevention and detection only);
  ∘ The level of systems (e.g., laws and policies).

▪ With the help of the filters, it will be possible to select interventions to respond to elder abuse that focus on preventing recurrence or those interventions to respond to elder abuse that focus on mitigating consequences.

#### 3.3.2 Columns in the framework

Columns in the framework will refer to elder abuse and the different sub‐types of elder abuse. Again, because this map includes studies on prevalence, consequences and risk and protective factors, and covers more than interventions, the columns will not strictly speaking refer to ʻoutcomes’, as is customary in maps of interventions. For instance, in relation to prevalence, elder abuse and its sub‐types (in the columns), will refer to the type of abuse being measured in the prevalence study in either the community or in institutions (in the rows). In relation to consequences, elder abuse and its sub‐types (in the columns), will be the predictors or ʻcauses’ and the different consequences the ʻeffect’ (in the rows). In the case of risk and protective factors, elder and its sub‐types (in the columns), will be the outcome predicted by the different types of risk and protective factors (in the rows). In relation to interventions, elder abuse and its sub‐types (in the columns) will be the outcome the different interventions are seeking to prevent, detect and respond to.

The following types of abuse will be included in the columns as 'outcomes' (see Supporting Information Appendix 1 for definitions):

- Any elder abuse
- Physical abuse
- Psychological abuse
- Sexual abuse
- Financial/material abuse
- Neglect
- Systemic/organizational abuse
- Poly‐victimization
- Other forms of abuse
- Potential adverse and unintended outcomes (for interventions only).

#### 3.3.3 Filtering variables

The following filters will allow sub‐groups of systematic reviews included in the map to be selected.

Filtering variables will include:

- The quality of the review;
- The type of synthesis (e.g., narrative, qualitative, mixed methods, or meta‐analytical);
- Online or face‐to‐face abuse (review focusing on online abuse, face‐to‐face‐abuse, or both; only applies to sexual, psychological, or financial/material abuse);
- Setting (review focusing on elder abuse in the community or in institutions or both in the community and in institutions);
- Source of report, mainly for reviews on prevalence (review includes studies that focus exclusively on reports by older person, trusted other, or service provider/staff);
- Victim characteristics (review focusing exclusively on a particular age sub‐group, a particular sex, people with a physical disability, or people with cognitive impairment, or other sub‐group);
- Perpetrator characteristics (reviews focusing exclusively on abuse by spouses/partners, staff in institutions, or other residents in institutions);
- WHO‐defined geographical region (review includes at least one study from that region);
- World Bank‐defined country income level (review includes at least one study from a country at that income level);
- Conflict of interest (declaration of a conflict of interest).

### 3.4 Eligibility criteria

#### 3.4.1 Types of study designs

Only systematic reviews and evidence and gap maps will be eligible for inclusion. We will follow the Campbell Collaboration's definition of systematic reviews—https://www.campbellcollaboration.org/what‐is‐a‐systematic‐review.html

Reviews which do not fit this definition of systematic review will be excluded.

Systematic reviews on the following aspects of elder abuse, in both the community and in institutions, will be eligible: prevalence, consequences, risk and protective factors and interventions to prevent, detect and respond to elder abuse. Systematic review which address more than one of these four aspects simultaneously or which address one or more of these four aspects as well as other aspects of elder abuse will also be included.

Detailed eligibility criteria are described in Supporting Information Appendix 4.

#### 3.4.2 Treatment of qualitative research

Systematic reviews including some or only qualitative studies will be included, as will qualitative primary studies included in systematic reviews.

#### 3.4.3 Types of settings

All the main types of settings in all regions of the world and in all country income levels in which elder abuse is likely to be recorded and in which interventions for elder abuse are likely to be implemented will be included. In practice, these will be divided into two main settings: community and institutional care settings.

#### 3.4.4 Status of studies

On‐going systematic reviews and EGMs will be included. Authors will be contacted to check if and when they will be completed and will be included or excluded accordingly. If excluded we will aim to include them in updates of the mega‐map provided they have been completed by then.

### 3.5 Search strategy

Systematic literature searches will be conducted on a wide range of databases without language restriction covering health and related disciplines such as social sciences, social care and psychology, and databases indexing research specific to older people. Databases will include (but not be limited to); MEDLINE, ASSIA, SOCIndex, PsycINFO, AgeLine, the Cochrane Database of Systematic Reviews, and the Campbell web site. Searches for subject headings (where available) will be combined with controlled vocabulary and free text terms relating to the population (older people), terms relating to types of elder abuse and terms relating to types of systematic reviews. Methodological search filters will be applied where possible to identify systematic reviews. The proposed search strategy has been peer‐reviewed by two senior information specialists. An example of a complete search strategy can be found in Supporting Information Appendix 3.

Grey literature will be identified by searching repositories, relevant organizational websites, dissertation and theses databases, databases of conference abstracts, and academic search engines such as Google Scholar. Key journal websites will also be searched. Reference lists of included reviews will be checked for any additional relevant studies. In addition, we will contact relevant individuals and organizations for information about unpublished or ongoing studies.

Search results will be imported into an Endnote library and duplicates will be removed. The references will then be imported into EPPI‐Reviewer for screening, data extraction and quality assessment.

### 3.6 Databases and other sources

Electronic databases

**Table:** 

CINAHL (Ebsco)
MEDLINE (Ebsco)
Embase.com
ASSIA (ProQuest)
PsycINFO (EBSCOhost)
Scielo
Abstracts in social gerontology (Ebsco)
AgeLine (Ebsco)
Socindex (Ebsco)
Social Work Abstracts (Ebsco)
Sociological Abstracts (Proquest)
Sociology Database (Proquest)
Science and Social Sciences Citation Indexes via Web of Science
CNKI (https://www.cnki.net/)
CBM (http://www.sinomed.ac.cn/)
WANFANG (https://www.wanfangdata.com.cn/index.html)
VIP (http://www.cqvip.com/)
Taiwan Academic Literature Database (https://www.airitilibrary.com/)
Hong Kong Chinese Journal Papers Index (http://hkinchippub.lib.cuhk.edu.hk/)
Index of Chinese periodical papers in macau (https://library.um.edu.mo/lib_homepage_en)

Grey Literature and other non‐standard database searches

**Table:** 

Proquest Dissertations & Theses
Cochrane Library
Scopus
LENUS—Irish health repository
Google Scholar
RIAN
The Irish LongituDinal Study on Ageing (TILDA)
Open Grey (Archive) at: OpenGrey—EASY (knaw.nl)
OAIster
BASE: https://www.base‐search.net/
GRAFT GRAFT: search across all the world's academic repositories. (jurn.org)
OpenAIRE | Find and Share research
Campbell Collaboration
Core Repository
Journal of Elder Abuse and Neglect
WHO Global Health Library https://www.globalindexmedicus.net/
JBI Systematic Review Register:
systematic‐review‐register—Systematic Review Register | Joanna Briggs Institute (jbi.global)

Websites from key organizations

**Table:** 

World Health Organization: https://www.who.int/
UNDESA: https://www.un.org/en/desa or https://www.un.org/development/desa/ageing/
OHCRH: https://www.ohchr.org/EN/pages/home.aspx
UN‐ECE: https://www.unece.org/info/ece‐homepage.html
UNFPA: https://www.unfpa.org/
UN women: https://www.unwomen.org/en
World Bank: https://www.worldbank.org/
Inter‐American Development Bank: https://www.iadb.org/en/about‐us/overview
Global Alliance of National Human Rights Institutions (GANHRI): https://ganhri.org/
Regional NHRI networks and the cross regional fora African Union: https://ijrcenter.org/national‐human‐rights‐institutions/regional‐nhri‐networks‐and‐forums/
EU: https://europa.eu/
Age Platform Europe: https://www.age‐platform.eu/
INPEA: http://www.inpea.net/
ASEAN: https://asean.org/
African Union: https://au.int/
Canadian Network for the Prevention of Elder Abuse: https://cnpea.ca/en/
HelpAge International: https://www.helpage.org/
Global Alliance for the Rights of Older People: https://www.eldis.org/organisation/A66465
US National Centre for Elder Abuse: https://ncea.acl.gov/
US Centers for Disease Control and Prevention: https://www.cdc.gov/
AARP International: https://www.aarpinternational.org/
US Department of Justice Elder Justice Initiative
https://www.justice.gov/elderjustice
US National Indigenous Elder Justice Initiative
https://www.nieji.org/
US National Adult Protective Services Association
https://www.napsa‐now.org/
International Association of Gerontology and Geriatrics: https://www.iagg.info/
NGO Committee on Ageing: http://www.ngocoa‐ny.org/
International Longevity Alliance: http://longevityalliance.org/
International Federation on Ageing: https://ifa.ngo/
Age UK | The UK's leading charity helping every older person who needs us
Age International | Helping older people live better lives
Centre for Ageing Better | Action today for all our tomorrows (ageing‐better.org.uk)
Hourglass (wearehourglass.org)
Prospero
Age Concern NZ
https://www.ageconcern.org.nz/Public/Information/Research/Elder_Abuse
National Institute on Aging
ResearchGate

### 3.7 Screening and selection of studies

All titles, abstracts and then full text of retrieved papers will be double screened, with a third‐party arbitrator in the event of disagreement. A data extraction form (with detailed definitions), detailed eligibility criteria and a screening tool—see Supporting Information Appendices 1, 4 and 5—have been developed and pilot tested for screening studies relevant to each of the four components of the mega‐map—prevalence, consequences, risk and protective factors, and interventions.

Only primary studies included in the systematic reviews will be included. Should these primary studies include multiple reports on the same study, the unit of analysis for the mega‐map will be the underlying study (i.e. sample), and not the multiple reports.

### 3.8 Data extraction, coding and management

Data extraction and coding will be done independently by two coders, with a third‐party arbitrator in the event of disagreement, using EPPI‐Reviewer.

Within the cells of the interactive map, the symbols used to represent the included reviews and primary studies included in the reviews will be designed so they can be changed in size and/or colour to explore the secondary dimensions described above.

The coded data will be converted to a JSON file, and EPPI‐Mapper will be used to generate the interactive map (https://eppi.ioe.ac.uk/cms/er4/EPPI‐Mapper/tabid/3790/Default.aspx).

### 3.9 Quality appraisal

The quality of the included systematic reviews will be assessed using AMSTAR 2—see Supporting Information Appendix 6, with a third‐party arbitrator in the event of disagreement. The AMSTAR‐2 results will be included in the EPPR‐Reviewer.

### 3.10 Ethical considerations

We will identify and extract information on conflicts of interest, when it is available, following the approach outlined in the Cochrane Handbook (https://training.cochrane.org/handbook/current/chapter‐07) and, should it become available, use the Tool for Addressing Conflicts of Interest in Trials (https://tacit.one/).

### 3.11 Analysis and presentation

#### 3.11.1 Unit of analysis

Each entry in the map will be a systematic review. The accompanying mega‐map report will identify the number of systematic reviews covered by the map

#### 3.11.2 Presentation

In addition to interventions and outcomes, as described in 3.3.1 and 3.3.2, filtering variables will be coded, as described in 3.3.3.

#### 3.11.3 Planned analysis

Two online maps will be produced:

1. A matrix with the prevalence, consequence, risk and protective factors and interventions categories and sub‐categories in rows (see 3.3.1) and outcome categories in columns (see 3.3.2), additional dimensions as filters (see 3.3.3), and size and colour of bubbles in cells indicating number of and quality of systematic reviews.
2. A map of the world showing distribution of primary studies by country and region hyperlinked to the abstract of the study itself.

A report on the map will also be developed which will provide tabulations and graphs of the publication of systematic reviews and primary studies included in them over time and the number and distribution of studies across the categories and sub‐categories in the rows of the framework (i.e. prevalence, consequences, risk and protective factors and interventions to prevent, detect and respond to elder abuse, see 3.3.1), and columns in the framework (types of elder abuse, see 3.3.1), and filtering variables (see 3.3.3), with accompanying narrative descriptions. The report will also include an identification of the main gaps.

### 3.12 Stakeholder engagement

The proposed framework was developed through a consultative process. An advisory and a stakeholders' group was formed made up of key experts on elder abuse from academia, international organizations, non‐governmental organizations and government working in the areas of research, policy, advocacy and practice.

A stakeholders meeting took place in February 2021 which convened some 35 members of the advisory and stakeholders' group. Its main aim was to consult stakeholders on the scope of the mega‐map and the categories and sub‐categories used for the framework of map—in the rows, columns and filter variables. The proposed framework is based on feedback received during this meeting, as well as further feedback received from members of the advisory group. See Supporting Information Appendix 7 for the report on the stakeholders meeting.

A second stakeholder meeting is planned once a draft map is available to get stakeholders' feedback.

## CONTRIBUTIONS OF AUTHORS

**Dr. Christopher Mikton**

Dr. Mikton will be providing the overall leadership and management for this project, including the development of the Title Registration Form, the Protocol and the final Report.

Content expertise: Focal point for elder abuse for WHO's Prevention of Violence Unit for 8.5 years;

Methodological expertise: Has carried out and helped carry out some 12 systematic reviews, review of reviews and meta‐analyses on various areas of violence prevention, including a series of three systematic reviews on the prevalence of elder abuse, led by Dr. Yon. Has never conducted a mega‐map or evidence and gap map before.

Information retrieval: Has conducted searches, screening and data extraction for several systematic reviews.

**Professor Marie Beaulieu**

Content expertise: Has been leading research project on elder abuse and neglect since 1987. She is the Chairholder of the Research Chair on Mistreatment of Older Adults, at the University of Sherbrooke. This Chair is financed by the Quebec Government.

Methodological expertise: Mainly a qualitative researcher but has also conducted programme development, implementation and evaluation. Expert on content for policy development with the Québec, Canada, France and Belgium Government.

Information retrieval: Has published several articles—including one systematic review, book chapters and books on the state of knowledge based on original data and searches and screening of available information.

**Dr. Yongjie Yon:**

Content expertise: Has over 10 years of experience conducting research on abuse over the life course including elder abuse, violence against women and child maltreatment.

Methodological expertise: Trained as a quantitative researcher. Has conducted three systematic reviews and meta‐analyses on elder abuse.

Information retrieval: Has published in leading journals on research relating to public health including elder abuse, ageism, violence prevention, intergenerational relations, oral health, housing and health disparities.

**Professor Amanda Phelan**

Content expertise: Her main research area is elder abuse and was Deputy Director of the Irish National Centre for the Protection of Older People, University College Dublin, 2014‐2020 and is currently a Director in Safeguarding Ireland. Has been involved in multiple research groups related to elder abuse, missed care, community nursing and person‐centred care. Her primary area of research is focused on safeguarding older people and older person care.

Methodological expertise: Has carried out several systematic reviews on older person care and a critical review of elder abuse screening tools in the Irish context.

Information retrieval: Has published multiple articles and book chapters on the state of knowledge based on searches and screening of available information. Has also edited two elder abuse books.

**Jessica Eustace‐Cook**

Information retrieval: Research Support Librarian, Library

**Julien Cadieux Genesse**

Content expertise: Has been a member of the Research Chair on Mistreatment of Older Adults team since 2017 and is its coordinator since 2019, at the University of Sherbrooke.

Methodological expertise: Has written research funding submissions with Marie Beaulieu financed by the Social Sciences and Humanities Research Council of Canada, by the Quebec Government, and the Department of Justice Canada. Held qualitative interviews and analyses on different projects led by Marie Beaulieu.

Information retrieval: Has published several articles and book chapters on the state of knowledge based on searches and screening of available information with Marie Beaulieu.

**Kevin St‐Martin**

Content expertise: Has been a member of the Research Chair on Mistreatment of Older Adults team at the University of Sherbrook since 2018 as a research assistant.

Methodological expertise: Held qualitative interviews and analyses in the context of his ongoing Master thesis in Social Work. Has carried out two scoping reviews his Master thesis.

Information retrieval: Has published several articles and book chapters on the state of knowledge on elder abuse and has done multiple databases search in the context of different projects led by Marie Beaulieu.

**Francis Lacasse**

Information retrieval: Librarain, Hôtel‐Dieu de Sherbrooke.

**Dr. Jennifer Storey**

Content experience: Has a background in psychology with 15 years of research and practice experience related to violence risk assessment and prevention as well as elder abuse and risk factors for perpetrators, victims and communities. She has developed a violence risk assessment tool for elder abuse.

Methodology expertise: Trained as a quantitative researcher. Her recent paper, Storey (2020), is the largest and most recent review on the risk factors for elder abuse. And she has completed other literature reviews.

Information retrieval: Has published in leading journals on research relating to elder abuse, ageism, violence prevention, violence risk and violence management.

**Dr. Michaela Rogers**

Content experience: Is a registered social worker and academic with nearly 30 years of research and practice experience in the domestic abuse sector. Latterly, her research and scholarship has included a focus on older adults in relation to interpersonal violence and abuse.

Methodology expertise: Qualitative researcher who has conducted systematic reviews.

Information retrieval: She has published in the field of gender‐based violence and has recently contributed to national guidance for social workers working with families affected by domestic abuse.

**Dr. Fiona Campbell**

Content experience: She has a background in public health and public health nursing.

Methodology expertise: Has over 20 years of experience in undertaking evidence synthesis to support decision making, policy and guideline development. She is an Editor with the Social Welfare Coordinating Group within the Campbell Collaboration. She has undertaken and supported the development of Cochrane and Campbell systematic reviews as well as for the WHO, NICE, NIHR, Alcohol Action, Public Health England and the Department of Health. She teaches a course in collaboration with the EPPI‐Centre on mapping reviews and developing Evidence Gap Maps. She has created evidence gap maps to support the development of guidelines and inform research priorities for Epilepsy Action and NIHR.

Information retrieval: Has published a large number of systematic reviews, she is the author of 12 Cochran reviews.

**Anthea Sutton**

Information retrieval: Information Resources Group Manager; Director of Professional Learning

**Dr. Parveen Ali**

Content experience: is a public health researcher with academic and research experience of over 20 years.

Methodology expertise: Dr. Ali is a mixed methods researcher and has extensive experience conducting systematic reviews and evidence synthesis of various topics and especially those related to elder abuse, gender‐based violence and domestic violence. She has extensive experience of creating face to face online courses that have used maps and other graphics to explain the extent and impact of abuse on victims.

Information retrieval: Published widely in the field of gender‐based violence and nursing.

**Dr. David Burnes**

Content experience: Has authored many peer‐reviewed publications and been awarded multiple external and federal level grants on the topic of elder abuse He received an NIH grant to conduct a longitudinal, population‐based elder abuse study on elder abuse risk factors and consequences. He led a paper to identify the prevalence and risk factors of elder abuse based on the New York State Elder Mistreatment Prevalence Study. Dr. Burnes has conducted several gerontology‐related systematic, meta‐analysis, or scoping reviews including on elder financial fraud and elder abuse intervention outcomes. Dr. Burnes has successfully contributed to projects with the WHO on topics related to elder abuse and ageism.

Methodology expertise: Systematic reviews, data synthesis

Information retrieval: Has published in leading journals on research relating to elder abuse, risk factors and consequences.

**Bridget Penhale**

Content experience: Is a registered social worker and academic. Her experience in elder abuse dates back to early work as a social worker in the 1980s. She published one of the first academic papers on elder abuse in the UK in 1993. Recognized nationally in the UK for her work on adult safeguarding/adult protection and internationally for her work on elder abuse. In 2010, she received the International Rosalie Wolf Award for her work in the field. She has undertaken research on adult safeguarding and elder abuse in the UK and Europe. Bridget has also acted as a consultant/advisor to the WHO, the Department of Health (in England and Scotland) and the European Commission in relation to elder abuse. She was also a member of an Expert Working Group on Violence Against Older Women convened by the UN.

Methodology expertise: Contributed to a number of gerontology‐related systematic and scoping reviews, including several related to elder abuse.

Information retrieval: Has published in leading journals on research relating to elder abuse.

**Professor Tova Band‐Winterstein**

Content experience: Research various aspects of elder abuse and neglect including intimate partner violence (IPV) in late life and elder self‐neglect. Recently, she has expanded her work on aging women who are sexually abused; older women who experienced incest in their early life; and IPV among multicultural groups. She is the chair of The Minerva Center on Intersectionality in Aging, and added research on special populations that are marginalized in a variety of ways, including abuse and neglect, but also reaching beyond this (e.g., lifelong disability, aging criminals).

Methodology expertise:

Information retrieval: She coordinated the first national survey on elder abuse and neglect in Israel and published two books on the topic: Like a wounded pigeon: Life stories of old battered women (2008) and Intimate violence across the lifespan: Interpersonal, familial and cross‐generational perspectives (2014).

**Professor Mark Lachs**

Content experience: Is a chronic disease epidemiologist who has conducted some of the largest longitudinal studies of elder abuse. Specifically, his work with the NIA EPEPE cohort demonstrated that being an elder abuse victim is associated with a threefold risk of death after adjustment for comorbidity and other factors. Subsequent work found that elder abuse was an independent predictor of nursing home placement. More recently his research has involved risk factors and outcomes for elder abuse occurring within long term care facilities between residents.

Methodology expertise: Is a trained quantitative researcher with expertise in longitudinal research.

**Information retrieval:** Has published in leading journals on research relating to public health including elder abuse.

**Professor Karl Pillemer**

Content experience: Is the most highly‐cited researcher in the field of elder abuse, having contributed to theory, research and intervention in the field over several decades. He conducted the first prevalence studies of the topic in family and institutional settings and the first large‐scale epidemiological survey of elder abuse and neglect, which established the benchmark prevalence rate for elder mistreatment. Since that time, he has conducted ground breaking work on risk factors for and consequences of elder abuse. Dr. Pillemer is also involved actively in intervention research and in translational research on elder abuse, exploring ways to speed the transfer of findings from basic research into scientifically tested interventions to prevent and treat elder abuse.

Methodology expertise: Has been involved in multiple systematic review projects involving elder abuse and related topics.

Information retrieval: Has published in leading journals on research relating to elder abuse and ageism.

**Kelly Marnfeldt** will be assisting with screening and data extraction. She is a doctoral student at the University of Southern California's Leonard Davis School of Gerontology.

Content expertise: Has conducted primary research on the prevention of mistreatment and neglect of older adults, and interventions to alleviate caregiver burden.

Methodological expertise: Trained in qualitative and mixed methods research. She has conducted literature reviews on the topics of caregiving, social isolation and loneliness, and elder mistreatment.

**Lilly Estenson** will be assisting with screening and data extraction. She is a doctoral student at the University of Southern California's Leonard Davis School of Gerontology.

Content expertise: Has a background in gerontological social work research and practice.

Methodological expertise: Trained in qualitative and mixed methods research with experience conducting systematic reviews.

**Dr. Mark Byrne** will be assisting with screening and data extraction. He is a research fellow at the School of Nursing and Midwifery, Trinity College Dublin.

Content expertise: Has experience in both qualitative and quantitative methods research. Conducted primary research (mixed methods) in the area of mental health.

Information retrieval: Has conducted literature reviews on the topics of inner conflict and depression.

## DECLARATIONS OF INTEREST

Yongjie Yon and Christopher Mikton have published several systematic reviews on the global prevalence of elder abuse (in the community, in institutions and among older women).

Marie Beaulieu has published several reports, articles, books chapters and books on the state of knowledge of elder abuse and neglect. She has also developed several practice tools. She has published original research on practices to prevent and respond to elder abuse and neglect. The Government of Quebec finances her Chair without a right to view and control her scientific production.

Julien Cadieux Genesse and Kevin St‐Martin, as co‐authors, published some reports, articles and books chapter with Marie Beaulieu.

Contributors Dr. Jennifer Storey, Dr. Michaela Rogers, Dr. Parveen Ali, Dr. David Burnes, Bridget Penhale, Professor Tova Band‐Winterstein, Professor Mark Lachs and Professor Karl Pillemer have published reports, articles and books chapters on the state of knowledge of elder abuse and neglect. This includes original research and reviews.

## PUBLISHED NOTES

None.

## SOURCES OF SUPPORT

 

### 1 INTERNAL SOURCES

- World Health Organization, Switzerland. Financial support
- University of Sherbrooke, Canada. Financial support

### 2 EXTERNAL SOURCES

- None.

## Supporting information

Supporting information.
