# Supplementary material for: PROTOCOL: Global elder abuse: A mega‐map of systematic reviews on prevalence, consequences, risk and protective factors and interventions
Source: Campbell Syst Rev. 2022 Apr 27;18(2):e1227. doi: 10.1002/cl2.1227 (PMC9046657; doi:10.1002/cl2.1227)
Supplement: Supplementary file 1 — Supporting information. [file CL2-18-e1227-s001.docx]

Appendices

1 Appendix 1: Data extraction form with definitions and examples of terms

[Enter te**Columns – elder abuse and types of elder abuse**

| Elder abuse | Types of elder abuse | Examples from studies |
| --- | --- | --- |
| *Elder abuse:*  A single or repeated act or lack of appropriate action, occurring within any relationship where there is an expectation of trust which causes harm or distress to an older person. | *Any abuse:* any of the types of abuse listed here, including “other”. |  |
|  | *Physical abuse:* the infliction of pain or injury, physical coercion, or physical or drug-induced restraint. | Push, pinch or scratch, slam against a wall, throw something at older person, burn or scald, pull hair, kick, shake, destroy property, throw or knock down. |
|  | *Psychological abuse:* the infliction of mental anguish. | Threaten to harm physically, insult or swear at, undermine and belittle, exclude or repeatedly ignore, prevent from seeing others, shout and yell, threaten to put in nursing home. |
|  | *Sexual abuse:* non-consensual sexual contact of any kind with the older person | Talk in sexual way, touch or try to touch in sexual way, make watch pornography, have or try to have sexual intercourse. |
|  | *Financial/material abuse:* the illegal or improper exploitation or use of funds or resources of the older person. | Take money or valuable possessions, force to give power of attorney, trick into making bad decision about finances, force to transfer ownership title, force to change will, use bank or credit card. |
|  | *Neglect:* the refusal or failure to fulfil a caregiving obligation. This may or may not involve a conscious and intentional attempt to inflict physical or emotional distress on the older person.  *Self-neglect* is not included in this definition and will not be included in this map. | Not receiving required help to shop for food or clothes, prepare meals, cut up and eat food, take medicine, wash and dress, get in and out of bed, use toilet. |
|  | *Systemic/organizational abuse – sometimes also referred to as organizational or institutional abuse:* rules, regulations, policies, or social practices that harm or discriminate against older adults. Systemic abuse includes rules that are developed for an apparently neutral purpose, but that hurt the person | Using physical restraints as an easy way to prevent falls; or diapering a person instead of helping them to the washroom, simply to save time or effort. |
|  | *Poly-victimization:* when a person aged 60 or older is harmed through multiple co-occurring or sequential types of elder abuse by one or more perpetrators, or when an older adult experiences one type of abuse perpetrated by multiple others with whom the older adult has a personal, professional or care recipient relationship in which there is a societal expectation of trust. | Co-occurring physical and financial abuse by the same family member. |
|  | *Other:* types of elder abuse not covered by the types above. |  |
|  | *Adverse or unintended outcomes*: An adverse outcome refers to any suboptimal outcome experienced by the patient. Unintended outcomes refer to outcomes – either positive ‘spill over’ effects or negative harms – not planned by those implementing an intervention. |  |

**Rows**

| Prevalence | Sub-categories | Examples from studies |
| --- | --- | --- |
| Prevalence: The proportion of adults 60+ who have experienced elder abuse in a given time period (any time period). | *Community:* an area, district, locality, neighbourhood, or vicinity where a group of people live. It includes private residences as well as locations that provide people with opportunities to work, engage in community life, receive services, etc. | Prevalence in the general population of community-dwelling older adults;  Prevalence among community-dwelling elders requiring assistance with ADLs;  Prevalence among community-dwelling elders receiving dementia care from informal caregivers. |
|  | *Institution or institutional care settings:* institutions in which long-term care is provided; these may include community centres, assisted living facilities, nursing homes, hospitals and other health facilities; institutional care setting is not defined only by the size of the facilities. | Prevalence in institutional settings (i.e., nursing homes, assisted living, residential care institutions, residential facilities, health facilities and skilled nursing facilities), at the national and subnational level (i.e., states/provinces, counties, districts and large cities) |

| Consequences | Sub-categories | Sub-sub-categories and examples from studies |
| --- | --- | --- |
| Consequences: The effects, both short- and long-term on individuals’ health and wellbeing and the societal costs of elder abuse. | *Service Use* | *Emergency departments*: physical injury, mental ill health episode; |
|  |  | *Hospitalization:* physical injury requiring hospitalization, mental health treatment, |
|  |  | *Social service referral or intervention*: Safeguarding and risk assessment, safety planning, care planning; |
|  |  | *GP visit* |
|  |  | *Call to police* |
|  |  | *Placement/admission of older person in supportive shelter:* domestic abuse shelter, older persons accommodation, safety planning. |
|  | *Mortality* | *Mortality – direct:* homicide;  *Mortality – indirect:* reduced life expectancy due longer-term consequences of abuse. |
|  | *Physical* | *Disability:* e.g., ADL and IADL, Other - mobility problems;  *Pain:* acute/chronic,  *General health:* General feeling of well-being, good immune system;  *Metabolic conditions:* Type 1 and type 2 diabetes;  *Digestive problems/gastrointestinal syndromes infective conditions, constipation*  *Weight problems:* increase or decrease/ nutritional and dietary consequences;  *STD / STIs:* urinary infections, chlamydia, genital herpes, gonorrhoea;  *Incontinence*  *Somatic complaints:* unexplained medical conditions. |
|  | *Mental health* | *Depression, anxiety, Sleep disturbance*  *Suicidal ideation, Attempted suicide, self-harm*  *OCD, PTSD*  *Stress*  *Other:* psychosis and other disorders, low mood, low frustration tolerance. |
|  | *Social & economic consequences* | *Placement of victim in institution:* loss of independence, financial cost to individual, institutional cost and resource implications; |
|  |  | *Social isolation and loneliness:* loss of social network, loss of independence, perpetrator controlling social connections; |
|  |  | *Social dysfunction:* lack of social connections resulting in withdrawal from family and communities, lack of confidence and self-esteem affecting social functioning; |
|  |  | *Loss of economic resources/poverty:* Perpetrator controlling finances, not allowed to seek employment, restricted access to money and resources, perpetrator does not allow victim to claim welfare benefits; |
|  |  | *Other:* Financial implications for institutions and care. |

| Risk and protective factors | Sub-categories | Sub-sub-categories and examples from studies |
| --- | --- | --- |
| Risk and protective factors that increase or reduces the risk of an older person, aged 60 years or over, experiencing abuse. The factors may relate to features of the individual, or the perpetrator or the environment. | Individual Victim | *Age group:* 60-80, 80+ (TBC) |
|  |  | *Gender:* M, F |
|  |  | *Ethnicity:* Ethnic majority, ethnic minority (TBC) |
|  |  | *Income- wealth:* Over or under poverty line in country (TBC) |
|  |  | *Marital status:* Marital status is the legally defined marital state. There are several types of marital status: single, married, widowed, divorced, separated and registered partnership |
|  |  | *Disability or physical health problems:* incontinence, chronic illness, poor health, recent decline in health, functional impairment, Activities of Daily Living (ADL); |
|  |  | *Dependency:* functional dependency, emotional dependency on the perpetrator, financial dependency on the perpetrator; |
|  |  | *Social isolation and loneliness:* has few social ties; rarely leaves home; is alone a lot or lonely; no trusted person, social support or emotional support; |
|  |  | *Mental health problem:* cognitive impairment, behavioural problems (difficult behaviour), mental health disorder; |
|  |  | *Alcohol and substance abuse:* physical or psychological problems due to substance use, substance us is affecting social adjustment (e.g., relationship problems, financial problems), substance use is impacting ability to protect self; |
|  |  | *Problems with stress and coping:* feels highly stress, upset, hopeless, anxious, unable to cope make decisions, is passively or avoidant coping. engaging in self-neglect; |
|  |  | *Education* |
|  |  | *Trauma or past abuse:* victim of previous abuse by perpetrator or other individual; |
|  |  | *Personality traits:* personality tests, scales etc. Personality disorders should go under mental health problem; |
|  |  | *Employment* |
|  |  | *Problems with attitudes:* minimizes perpetrator's behaviour, ambivalent about perpetrator behaviour, refusal to report perpetrator or behaviour; |
|  |  | *Lives with others*: lives with perpetrator, place is overcrowded or lacks privacy; |
|  |  | *Conflictual relationships with others or with perpetrator:* conflictual relationship with perpetrator pre or post the start of the abuse,conflict with others, arguments, severe unwarranted anger. |
|  | Individual Perpetrator | *Age group:* 60-80, 80+ (TBC) |
|  |  | *Gender:* M, F |
|  |  | *Ethnicity:* Ethnic majority, ethnic minority (TBC) |
|  |  | *Staff (type of staff):* less education, younger, less experience, less satisfied with job, see older adults as childlike, burnout, external stress, lack of empathy; |
|  |  | *Mental health problem:* cognitive impairments, mental disorder, personality disorder, homicidal or suicidal ideation; |
|  |  | *Alcohol and substance abuse*: physical or psychological problems due to substance use, substance us is affecting social adjustment (e.g. fired, criminal conviction, financial problems); |
|  |  | *Dependency:* Financial difficulties, unemployed, difficulty maintaining work, is not self-sufficient, is depending on others for finances. Emotionally or socially dependent on the victim; |
|  |  | *Personality traits:* look for personality tests, scales etc. Personality disorders should go under mental health problems; |
|  |  | *Caregiver inexperience:* unrealistic expectations of caregiving; |
|  |  | *Caregiving reluctance/giving up work:* unhappy with caregiving role, feels obligated, resentful of caregiving role; |
|  |  | *Caregiving burden or stress:* subjective (most important), objective; |
|  |  | *Problems with stress and coping*: sudden increase in stress, unable to cope with stress, unable to make decisions, feels highly stressed, unable to cope with day to day stress; |
|  |  | *Trauma or past abuse*: past witnessing or experience of abuse as a child or adolescent. Any type of abuse, physical, sexual, neglect; |
|  |  | *Physical Health problems*: chronic illness, poor health, recent decline in health, functional impairment, IADL, ADL; |
|  |  | *History of violence/behaviour problems and antisocial attitudes:* convictions, breaches of court orders, ageism, lack of empathy, hostility, poor impulse control, antisocial personality disorder; |
|  |  | *Problems with relationships:* conflictual relationships with others, mistreats others, is socially isolated, feels socially isolated, has little social support, feels has little social support. |
|  | Relationship | *Family disharmony, poor or conflictual relationships* |
|  |  | *Poor understanding/unrealistic expectations* |
|  |  | *Kinship:* children or partner; |
|  |  | *Financial dependence to older person* |
|  |  | *Functional dependency on the older* person (i.e. emotional) |
|  |  | *Others* |
|  | Social and economic factors | *Discrimination* (i.e. ageism, sexism, racism) |
|  |  | *Violent culture* (i.e. violent neighbourhood, high crime) |
|  |  | *Problems with accessing resources*: physical access is difficult, rurality, lack of transport |
|  |  | *Problems with affordability of resources*: high cost of care, treatment, limited financial resources |
|  |  | *lack of available community resources and support:* lack of care facilities and staff, lack of treatment facilities. lack of transition homes, social services do not offer enough support, family is unavailable assist. |
|  | Institutional characteristics | *Institutional tolerance of aggression* |
|  |  | *Poor or inadequate training of staff* |
|  |  | *lack of support for paid care staff* |
|  |  | *Poor management*: lack of abuse reporting procedures, inflexible routines and regimes, abrupt changes of rooms and environments, negligent hiring with limited or no screening; |
|  |  | *Lack safety resources*: failure to implement safety plans. |

| Interventions to prevent | Sub-categories | Examples from studies |
| --- | --- | --- |
| Intervention to prevent: organized effort to prevent elder abuse from occurring in the first place. | *Older people:* people 60 years old and over. | Pamphlet distribution by primary care physicians to all older persons;  The purple ribbon initiative during the world elder abuse awareness day. |
|  | *Professional caregiver (physicians, social workers, etc.):*  a [care] provider associated with a formal service system, whether a paid worker or a volunteer. | Professional training on how to properly interact with older people in the context of care. |
|  | *Non-professional caregiver (family, friend, etc.):*  any relative, partner, friend or neighbour who has a significant personal relationship with, and provides a broad range of assistance for, an older person or an adult with a chronic or disabling condition. | Interventions aimed at reducing caregiver burden. |
|  | *General population*:  every person who is not trained, qualified or experience in a particular subject or activity [elder abuse]. | Televised elder abuse awareness campaigns. |
|  | *Institution (long term care, hospitals, banks, etc.):*  any established organization or corporation, especially of a public character and any facility or establishment in which people live and receive care typically in a confined setting. | Implementation of an establishment policy to prevent elder abuse (presenting what abuse is and how to avoid it). |
|  | *System level*: interventions to prevent elder abuse at the level of organizations, policies, laws, and power structures. The focus is not directly on individuals and communities but on the systems that influence their health. | Adoption of social policy inclusive of older people;  Policies fighting against ageism;  Policies favouring intergenerational approaches. |

| Interventions to detect | Sub-categories | Examples from studies |
| --- | --- | --- |
| Intervention to detect: organized effort to identify whether elder abuse has occurred, is occurring, or if there is a high risk of elder abuse occurring in the future. | Older people | Providing auto-administered detection tools to older adults. |
|  | Professional caregiver | Improving primary care physician’s knowledge on how to identify cases of elder abuse. |
|  | Non-professional caregiver (family, friend, etc.) | Raising awareness of what elder abuse is and how to report it. |
|  | General population | Raising awareness of what elder abuse is and how to report it. |
|  | Institution (long term care, hospitals, banks, etc.) | Flagging suspicious financial transactions;  Have a system for filing incident reports. |
|  | System level (including laws and policies) | Mandatory reporting laws and guidelines on when to report. |

| Interventions to respond | Sub-categories | Examples from studies |
| --- | --- | --- |
| Intervention to respond: organized effort to either prevent the recurrence of elder abuse, focused on perpetrators, after it has occurred or mitigate the consequences of elder abuse, focused on the victim, after it has occurred. | Older people | Interventions removing the older person from an abusive situation. |
|  | Professional caregiver | Putting the person on leave pending an investigation. |
|  | Institution (long term care, hospitals, banks, etc.) | Educational interventions to reduce the use of physical restraint in institutions. |
|  | System level (including laws and policies) | Create new laws, change discriminating policies, etc. |

**Filtering variables**

| Category | Sub-category | Examples |  |
| --- | --- | --- | --- |
| Quality of systematic review | High | We will follow the method outline in this paper to distinguish between high, moderate, low, and critically low quality of systematic review: https://www.bmj.com/content/bmj/358/bmj.j4008.full.pdf |  |
|  | Moderate |  |  |
|  | Low |  |  |
|  | Critically low |  |  |
| Type of syntheses | Narrative syntheses: an approach to the systematic review and synthesis of findings from multiple quantitative studies that relies primarily on the use of words and text to summarise and explain the findings of the synthesis. |  |  |
|  | Qualitative syntheses: the systematic review and synthesis of individual qualitative studies. Different sub-types of qualitative syntheses include, for instance, meta-syntheses or ethnographic syntheses. |  |  |
|  | Mixed-methods synthesis: combining the findings of qualitative and quantitative studies within a single systematic review to address the same overlapping or complementary review questions. |  |  |
|  | *Meta-analytic syntheses:* syntheses which use a technique that statistically combines the results of quantitative studies to provide a more precise effect of the results. |  |  |
| Online or face-to-face abuse (applies to sexual, psychological, and financial abuse) | Online: abuse that occurs online (via a smart phone, tablet, computer, or other device)) |  |  |
|  | Face-to-face: abuse that occurs in person |  |  |
| Setting | *Community:* an area, district, locality, neighbourhood, or vicinity where a group of people live. | It includes private residences as well as locations that provide people with opportunities to work, engage in community life, receive services, etc. |  |
|  | *Institution or institutional care settings:* institutions in which long-term care is provided; institutional care setting is not defined only by the size of the facilities. | May include community centres, assisted living facilities, nursing homes, hospitals and other health facilities; |  |
| Source of report (prevalence only) | Older person/resident of institution | Person aged 60 or older living in an institution reporting a situation of abuse |  |
|  | *Trusted other (e.g. family):* any familial relations or relations where there is an expectation of trust |  |  |
|  | *Service provider/staff:* a [care] provider associated with a formal service system, whether a paid worker or a volunteer. | Care provider reporting a situation of abuse |  |
| Victim characteristics | Age group (60-79; 80+) |  |  |
|  | Sex: (male, female) |  |  |
|  | *Physical disability:* A physical disability is a physical condition that affects a person’s mobility, physical capacity, stamina, or dexterity. | Abuse of a person presenting a physical disability. This can include brain or spinal cord injuries, multiple sclerosis, cerebral palsy, respiratory disorders, epilepsy, hearing and visual impairments and more. |  |
|  | *Cognitive impairment*: when a person has trouble  remembering, learning new things, concentrating,  or making decisions that affect their everyday | Abuse of a person presenting a cognitive impairment that affects their everyday life. This can be caused by dementia. |  |
| Perpetrator-relationship | Spouse/Partner |  |  |
|  | Staff in institutions |  |  |
|  | Other residents in institutions |  |  |
| Country | Specify |  |  |
| Category | Sub-category | Full list of countries included |  |
| Geographical region (WHO-defined) | African Region | Algeria, Angola, Benin, Botswana, Burkina Faso, Burundi, Cabo Verde, Cameroon, Central African Republic, Chad, Comoros (the), Congo, Côte d'Ivoire, Democratic Republic of the Congo, Equatorial Guinea, Eritrea, Eswatini, Ethiopia, Gabon, Ghana, Guinea, Guinea-Bissau, Kenya, Lesotho, Liberia, Madagascar, Malawi, Mali, Mauritania, Mauritius, Mozambique, Namibia, Niger, Nigeria, Rwanda, Sao Tome and Principe, Senegal, Seychelles, Sierra Leone, South Africa, South Sudan, the Islamic Republic of the Gambia, Togo, Uganda, United Republic of Tanzania, Zambia, Zimbabwe |  |
|  | Region of the Americas | Antigua and Barbuda, Argentina, Bahamas (the), Barbados, Belize, Bolivia (Plurinational State of), Brazil, Canada, Chile, Colombia, Costa Rica, Cuba, Dominica, Dominican Republic (the), Ecuador, El Salvador, Grenada, Guatemala, Guyana, Haiti, Honduras, Jamaica, Mexico, Nicaragua, Panama, Paraguay, Peru, Saint Kitts and Nevis, Saint Lucia, Saint Vincent and the Grenadines, Suriname, Trinidad and Tobago, United States of America, Uruguay, Venezuela (Bolivarian Republic of) |  |
|  | South-East Asian Region | Bangladesh, Bhutan, Democratic People's Republic of Korea, India, Indonesia, Maldives, Myanmar, Nepal, Sri Lanka, Thailand, Timor-Leste |  |
|  | European Region | Albania, Andorra, Armenia, Austria, Azerbaijan, Belarus, Belgium, Bosnia and Herzegovina, Bulgaria, Croatia, Cyprus, Czech Republic, Denmark, Estonia, Finland, France, Georgia, Germany, Greece, Hungary, Iceland, Ireland, Israel, Italy, Kazakhstan, Kyrgyzstan, Latvia, Lithuania, Luxembourg, Malta, Monaco, Montenegro, Netherlands, North Macedonia, Norway, Poland, Portugal, Republic of Moldova, Romania, Russian Federation, San Marino, Serbia, Slovakia, Slovenia, Spain, Sweden, Switzerland, Tajikistan, Turkey, Turkmenistan, Ukraine, United Kingdom of Great Britain and Northern Ireland (the), Uzbekistan |  |
|  | Eastern Mediterranean Region | Afghanistan, Bahrain, Djibouti, Egypt, Iran (Islamic Republic of), Iraq, Jordan, Kuwait, Lebanon, Libya, Morocco, Oman, Pakistan, Qatar, Saudi Arabia, Somalia, Sudan, Syrian Arab Republic, Tunisia, United Arab Emirates, Yemen |  |
|  | Western Pacific Region | Australia, Brunei Darussalam, Cambodia, China, Cook Islands, Fiji, Japan, Kiribati, Lao People's Democratic Republic, Malaysia, Marshall Islands, Micronesia (Federated States of), Mongolia, Nauru, New Zealand, Niue, Palau, Papua New Guinea, Philippines, Republic of Korea, Samoa, Singapore, Solomon Islands, Tonga, Tuvalu, Vanuatu, Viet Nam |  |
| Country income level (World Bank-defined based on 2021)  **Group**  Low income  Lower-middle income  1,046 – 4,095  Upper-middle income  4,096 -12,695  High income  > 12,695 | High income country | https://datahelpdesk.worldbank.org/knowledgebase/articles/906519-world-bank-country-and-lending-groups |  |
|  | Upper middle income country |  |  |
|  | Lower middle income country |  |  |
|  | Low income country |  |  |
|  |  |  |  |
| Conflict of interest | A *conflict of interest* refers to a set of circumstances that creates a risk that professional judgment or actions regarding a primary interest will be unduly influenced by a secondary interest. | With be taken from declaration of conflict of interest in the review, following approach described here: https://training.cochrane.org/handbook/current/chapter-07#section-7-8 |  |

## 2 Appendix 2: Detailed framework

|  |  | Any abuse | Physical | Psychological | Sexual | Financial/material | Neglect | Systemic/organizational abuse | Polyvictimization | Other | Potential adverse or unintended outcomes (for interventions studies) |  |
| --- | --- | --- | --- | --- | --- | --- | --- | --- | --- | --- | --- | --- |
| Prevalence |  |  |  |  |  |  |  |  |  |  |  |  |
| Prevalance | Community settings |  |  |  |  |  |  |  |  |  |  |  |
|  | Institutional settings |  |  |  |  |  |  |  |  |  |  |  |
| Consequences |  |  |  |  |  |  |  |  |  |  |  | Note: more than one consequence can apply |
| Service Use | Emergency departments |  |  |  |  |  |  |  |  |  |  |  |
|  | Hospitalization |  |  |  |  |  |  |  |  |  |  |  |
|  | Social service referral or intervention |  |  |  |  |  |  |  |  |  |  |  |
|  | GP visit |  |  |  |  |  |  |  |  |  |  |  |
|  | Call to police |  |  |  |  |  |  |  |  |  |  |  |
|  | Placement/admission of older person in supportive shelter |  |  |  |  |  |  |  |  |  |  |  |
| Mortality | Mortality - direct |  |  |  |  |  |  |  |  |  |  |  |
|  | Mortality - indirect |  |  |  |  |  |  |  |  |  |  |  |
| Physical | Disability |  |  |  |  |  |  |  |  |  |  |  |
|  | Pain |  |  |  |  |  |  |  |  |  |  |  |
|  | General health |  |  |  |  |  |  |  |  |  |  |  |
|  | Metabolic conditions |  |  |  |  |  |  |  |  |  |  |  |
|  | Digestive problems/gastrointestinal syndromes  syndromes |  |  |  |  |  |  |  |  |  |  |  |
|  | Weight problems |  |  |  |  |  |  |  |  |  |  |  |
|  | STD / STIs |  |  |  |  |  |  |  |  |  |  |  |
|  | Incontinence |  |  |  |  |  |  |  |  |  |  |  |
|  | Somatic complaints |  |  |  |  |  |  |  |  |  |  |  |
|  | Other - mobility problems |  |  |  |  |  |  |  |  |  |  |  |
| Mental health | Depression |  |  |  |  |  |  |  |  |  |  |  |
|  | Anxiety |  |  |  |  |  |  |  |  |  |  |  |
|  | Sleep |  |  |  |  |  |  |  |  |  |  |  |
|  | Suicidal ideation |  |  |  |  |  |  |  |  |  |  |  |
|  | OCD |  |  |  |  |  |  |  |  |  |  |  |
|  | Attempted suicide |  |  |  |  |  |  |  |  |  |  |  |
|  | self harm |  |  |  |  |  |  |  |  |  |  |  |
|  | Stress |  |  |  |  |  |  |  |  |  |  |  |
|  | PTSD |  |  |  |  |  |  |  |  |  |  |  |
|  | Other |  |  |  |  |  |  |  |  |  |  |  |
| Social & economic consequences | Placement of victim in institution |  |  |  |  |  |  |  |  |  |  |  |
|  | Social isolation and loneliness |  |  |  |  |  |  |  |  |  |  |  |
|  | Social dysfunction |  |  |  |  |  |  |  |  |  |  |  |
|  | Loss of economic resources/poverty |  |  |  |  |  |  |  |  |  |  |  |
|  | Other |  |  |  |  |  |  |  |  |  |  |  |
| Other | Self-rated health |  |  |  |  |  |  |  |  |  |  |  |
|  | Other |  |  |  |  |  |  |  |  |  |  |  |
| Risk and protective factors |  |  |  |  |  |  |  |  |  |  |  |  |
| Risk factors |  |  |  |  |  |  |  |  |  |  |  |  |
| Individual Victim | Age group |  |  |  |  |  |  |  |  |  |  |  |
|  | Gender |  |  |  |  |  |  |  |  |  |  |  |
|  | Ethnicity |  |  |  |  |  |  |  |  |  |  |  |
|  | Income- wealth |  |  |  |  |  |  |  |  |  |  |  |
|  | Marital status |  |  |  |  |  |  |  |  |  |  |  |
|  | Disability or physical health problems |  |  |  |  |  |  |  |  |  |  |  |
|  | Dependency |  |  |  |  |  |  |  |  |  |  |  |
|  | Problems with stress and coping |  |  |  |  |  |  |  |  |  |  |  |
|  | Education |  |  |  |  |  |  |  |  |  |  |  |
|  | Mental health problems (including cognitive impairment) |  |  |  |  |  |  |  |  |  |  |  |
|  | Trauma or past abuse |  |  |  |  |  |  |  |  |  |  |  |
|  | Personality traits |  |  |  |  |  |  |  |  |  |  |  |
|  | Employment |  |  |  |  |  |  |  |  |  |  |  |
|  | Problems with attitudes |  |  |  |  |  |  |  |  |  |  |  |
|  | Lives with others |  |  |  |  |  |  |  |  |  |  |  |
|  | Conflictual relationships with others or with perpetrator |  |  |  |  |  |  |  |  |  |  |  |
|  | Other specify: |  |  |  |  |  |  |  |  |  |  |  |
| Individual Perpetrator | Age group |  |  |  |  |  |  |  |  |  |  |  |
|  | Gender |  |  |  |  |  |  |  |  |  |  |  |
|  | Ethnicity |  |  |  |  |  |  |  |  |  |  |  |
|  | Staff (type of staff) |  |  |  |  |  |  |  |  |  |  |  |
|  | Mental health problem (including cognitive impairment) |  |  |  |  |  |  |  |  |  |  |  |
|  | Alcohol and substance abuse |  |  |  |  |  |  |  |  |  |  |  |
|  | Dependency |  |  |  |  |  |  |  |  |  |  |  |
|  | Personality traits |  |  |  |  |  |  |  |  |  |  |  |
|  | Caregiver inexperience |  |  |  |  |  |  |  |  |  |  |  |
|  | Caregiving reluctance/giving up work |  |  |  |  |  |  |  |  |  |  |  |
|  | Caregiving burden or stress |  |  |  |  |  |  |  |  |  |  |  |
|  | Problems with stress and coping |  |  |  |  |  |  |  |  |  |  |  |
|  | Trauma or past abuse |  |  |  |  |  |  |  |  |  |  |  |
|  | Physical health problems |  |  |  |  |  |  |  |  |  |  |  |
|  | History of violence/behaviour problems and antisocial attitudes |  |  |  |  |  |  |  |  |  |  |  |
|  | Problems with relationships |  |  |  |  |  |  |  |  |  |  |  |
|  | Other specify: |  |  |  |  |  |  |  |  |  |  |  |
| Relationship | Family disharmony, poor or conflictual relationships |  |  |  |  |  |  |  |  |  |  |  |
|  | Poor understanding/unrealistic expectations |  |  |  |  |  |  |  |  |  |  |  |
|  | Kinship: children or partner |  |  |  |  |  |  |  |  |  |  |  |
|  | Financial dependence on older person |  |  |  |  |  |  |  |  |  |  |  |
|  | Functional dependency on the older person (i.e. emotional) |  |  |  |  |  |  |  |  |  |  |  |
|  | Other specify: |  |  |  |  |  |  |  |  |  |  |  |
| Community & society | Low social support |  |  |  |  |  |  |  |  |  |  |  |
|  | Living with others/shared living arrangment |  |  |  |  |  |  |  |  |  |  |  |
|  | Discrimination (i.e. ageism, sexism, racism) |  |  |  |  |  |  |  |  |  |  |  |
|  | Social and economic factors |  |  |  |  |  |  |  |  |  |  |  |
|  | Violent culture (i.e. violent neighbourhood, high crime) |  |  |  |  |  |  |  |  |  |  |  |
|  | Problems with accessing resources |  |  |  |  |  |  |  |  |  |  |  |
|  | Problems with affordability of resources |  |  |  |  |  |  |  |  |  |  |  |
|  | Lack of available community resources and support |  |  |  |  |  |  |  |  |  |  |  |
|  | Other specify: |  |  |  |  |  |  |  |  |  |  |  |
| Institutional characteristics | Institutional tolerance of aggression |  |  |  |  |  |  |  |  |  |  |  |
|  | Poor or inadequate training of staff |  |  |  |  |  |  |  |  |  |  |  |
|  | lack of support for paid care staff |  |  |  |  |  |  |  |  |  |  |  |
|  | Poor management |  |  |  |  |  |  |  |  |  |  |  |
|  | Lack safety resources |  |  |  |  |  |  |  |  |  |  |  |
|  | Other specify: |  |  |  |  |  |  |  |  |  |  |  |
| Protective factors |  |  |  |  |  |  |  |  |  |  |  |  |
| Indvidual perpetrator | Other specify: |  |  |  |  |  |  |  |  |  |  |  |
| Individual victim | Social support |  |  |  |  |  |  |  |  |  |  |  |
|  | Resilience |  |  |  |  |  |  |  |  |  |  |  |
|  | Other specify: |  |  |  |  |  |  |  |  |  |  |  |
| Relationship | Other specify: |  |  |  |  |  |  |  |  |  |  |  |
| Community & society | Other specify: |  |  |  |  |  |  |  |  |  |  |  |
| Intitutional characteristics | Caring staff |  |  |  |  |  |  |  |  |  |  |  |
|  | Well-paid staff |  |  |  |  |  |  |  |  |  |  |  |
|  | Other specify: |  |  |  |  |  |  |  |  |  |  |  |
| Interventions -Prevention |  |  |  |  |  |  |  |  |  |  |  |  |
| Prevention | Older people |  |  |  |  |  |  |  |  |  |  |  |
|  | Professional caregiver |  |  |  |  |  |  |  |  |  |  |  |
|  | Non-professional caregiver (family, friend, etc.) |  |  |  |  |  |  |  |  |  |  |  |
|  | General population |  |  |  |  |  |  |  |  |  |  |  |
|  | Institution (long term care, hospitals, banks, etc.) |  |  |  |  |  |  |  |  |  |  |  |
|  | System level (including laws and policies) |  |  |  |  |  |  |  |  |  |  |  |
| Interventions - Detection |  |  |  |  |  |  |  |  |  |  |  |  |
| Detection | Older people |  |  |  |  |  |  |  |  |  |  |  |
|  | Professional caregiver |  |  |  |  |  |  |  |  |  |  |  |
|  | Non-professional caregiver (family, friend, etc.) |  |  |  |  |  |  |  |  |  |  |  |
|  | General population |  |  |  |  |  |  |  |  |  |  |  |
|  | Institution (long term care, hospitals, banks, etc.) |  |  |  |  |  |  |  |  |  |  |  |
|  | System level (including laws and policies) |  |  |  |  |  |  |  |  |  |  |  |
| Interventions - Reponse |  |  |  |  |  |  |  |  |  |  |  |  |
| Response | Older people |  |  |  |  |  |  |  |  |  |  |  |
|  | Professional caregiver |  |  |  |  |  |  |  |  |  |  |  |
|  | Non-professional caregiver (family, friend, etc.) |  |  |  |  |  |  |  |  |  |  |  |
|  | Institution (long term care, hospitals, banks, etc.) |  |  |  |  |  |  |  |  |  |  |  |
|  | System level (including laws and policies) |  |  |  |  |  |  |  |  |  |  |  |
|  |  |  |  |  |  |  |  |  |  |  |  |  |
|  |  |  |  |  |  |  |  |  |  |  |  |  |
|  |  |  |  |  |  |  |  |  |  |  |  |  |
| Filters | |  |  |  |  |  |  |  |  |  |  |  |
| Quality of systematic review | High |  |  |  |  |  |  |  |  |  |  |  |
|  | Moderate |  |  |  |  |  |  |  |  |  |  |  |
|  | Low |  |  |  |  |  |  |  |  |  |  |  |
|  | Critically low |  |  |  |  |  |  |  |  |  |  |  |
| Type of syntheses | Narrative syntheses |  |  |  |  |  |  |  |  |  |  |  |
|  | Qualitative syntheses |  |  |  |  |  |  |  |  |  |  |  |
|  | Mixed-method synthesis |  |  |  |  |  |  |  |  |  |  |  |
|  | Meta-analytic syntheses |  |  |  |  |  |  |  |  |  |  |  |
| Online or face-to-face abuse (applies to sexual, psychological, and financial abuse) | Online |  |  |  |  |  |  |  |  |  |  |  |
|  | Face-to-face |  |  |  |  |  |  |  |  |  |  |  |
|  | Both online and face-to-face |  |  |  |  |  |  |  |  |  |  |  |
|  | Not specified |  |  |  |  |  |  |  |  |  |  |  |
| Setting | Community |  |  |  |  |  |  |  |  |  |  |  |
|  | Institutional |  |  |  |  |  |  |  |  |  |  |  |
|  | Both community and institutional |  |  |  |  |  |  |  |  |  |  |  |
| Source of report (prevalence only) | Older person/resident of institution |  |  |  |  |  |  |  |  |  |  |  |
|  | Trusted other (e.g. family) |  |  |  |  |  |  |  |  |  |  |  |
|  | Service provider/staff |  |  |  |  |  |  |  |  |  |  |  |
|  | Authorities (e.g. Adult Protective Services) |  |  |  |  |  |  |  |  |  |  |  |
|  | Unclear |  |  |  |  |  |  |  |  |  |  |  |
|  | Other, specify: |  |  |  |  |  |  |  |  |  |  |  |
| Victim characteristics | Age group |  |  |  |  |  |  |  |  |  |  |  |
|  | Sex |  |  |  |  |  |  |  |  |  |  |  |
|  | Physical disability |  |  |  |  |  |  |  |  |  |  |  |
|  | Cognitive impairment |  |  |  |  |  |  |  |  |  |  |  |
|  | Other |  |  |  |  |  |  |  |  |  |  |  |
| Perpetrator-relationship | Spouse/Partner |  |  |  |  |  |  |  |  |  |  |  |
|  | Children |  |  |  |  |  |  |  |  |  |  |  |
|  | Other family member |  |  |  |  |  |  |  |  |  |  |  |
|  | Non-family caregiver (formal or informal) |  |  |  |  |  |  |  |  |  |  |  |
|  | Staff in institutions |  |  |  |  |  |  |  |  |  |  |  |
|  | Other residents in institutions |  |  |  |  |  |  |  |  |  |  |  |
|  | Telemarketer (or other person on telephone or online) |  |  |  |  |  |  |  |  |  |  |  |
|  | Other |  |  |  |  |  |  |  |  |  |  |  |
| Region (WHO-defined) | African Region |  |  |  |  |  |  |  |  |  |  |  |
|  | Region of the Americas |  |  |  |  |  |  |  |  |  |  |  |
|  | South-East Asian Region |  |  |  |  |  |  |  |  |  |  |  |
|  | European Region |  |  |  |  |  |  |  |  |  |  |  |
|  | Eastern Mediterranean Region |  |  |  |  |  |  |  |  |  |  |  |
|  | Western Pacific Region |  |  |  |  |  |  |  |  |  |  |  |
|  | Not specified |  |  |  |  |  |  |  |  |  |  |  |
| Country income level (World Bank-defined) | High income country |  |  |  |  |  |  |  |  |  |  |  |
|  | Upper middle income country |  |  |  |  |  |  |  |  |  |  |  |
|  | Lower middle income country |  |  |  |  |  |  |  |  |  |  |  |
|  | Low income country |  |  |  |  |  |  |  |  |  |  |  |
|  | Not specified |  |  |  |  |  |  |  |  |  |  |  |
| Conflict of interest | From declaration of conflict of interest in review |  |  |  |  |  |  |  |  |  |  |  |

## 3 Appendix 3: Search strategy

*Search strategy from MEDLINE via EBSCO*

| **#** | **Query** | **Limiters/Expanders** | **Last Run Via** | **Results** |
| --- | --- | --- | --- | --- |
| S1 | (MH "Systematic Reviews as Topic") OR (MH "Meta-Analysis as Topic+") OR (PT "Systematic Review") OR (PT "Meta-Analysis") | Expanders - Apply equivalent subjects  Search modes - Boolean/Phrase | Interface - EBSCOhost Research Databases  Search Screen - Advanced Search  Database - MEDLINE | 247,132 |
| S2 | (TI (systematic* N3 review*)) OR (AB (systematic* N3 review*)) OR (TI (systematic* N3 bibliographic*)) OR (AB (systematic* N3 bibliographic*)) OR (TI (systematic* N3 literature)) OR (AB (systematic* N3 literature)) OR (TI (comprehensive* N3 literature)) OR (AB (comprehensive* N3 literature)) OR (TI (comprehensive* N3 bibliographic*)) OR (AB (comprehensive* N3 bibliographic*)) OR (TI (integrative N3 review)) OR (AB (integrative N3 review)) OR (TI (qualitative N3 “evidence synthes*”)) OR (AB (qualitative N3 “evidence synthes*”)) OR (TI (qualitative N3 synthes*)) OR (AB (qualitative N3 synthes*)) OR ((JN “Cochrane Database of Systematic Reviews”) OR (JN “Joanna Briggs”) OR (JN “JBI”) OR (TI (information N2 synthesis)) OR (TI (data N2 synthesis)) OR (AB (information N2 synthesis)) OR (AB (data N2 synthesis)) OR (TI (data N2 extract*)) OR (AB (data N2 extract*)) OR (TI (medline OR pubmed OR psyclit OR cinahl OR (psycinfo not “psycinfo database”) OR “web of science” OR scopus OR embase)) OR (AB (medline OR pubmed OR psyclit OR cinahl OR (psycinfo not “psycinfo database”) OR “web of science” OR scopus OR embase)) OR (TI (meta-analy* OR metaanaly*)) OR (AB (meta-analy* OR metaanaly*)) OR (TI (meta-synthes* OR metasynthes*)) OR (AB (meta-synthes* OR metasynthes*)) | Expanders - Apply equivalent subjects  Search modes - Boolean/Phrase | Interface - EBSCOhost Research Databases  Search Screen - Advanced Search  Database - MEDLINE | 465,610 |
| S3 | S1 OR S2 | Expanders - Apply equivalent subjects  Search modes - Boolean/Phrase | Interface - EBSCOhost Research Databases  Search Screen - Advanced Search  Database - MEDLINE | 485,626 |
| S4 | AB ( ("old* person*" OR “old* people*” OR “old* individual*” OR aged OR gerontol* OR elder* OR geriatric* OR ageing OR aging OR senior* OR retir* OR octogenarian* OR nonagenarian* OR septuagenarian* OR sexagenarian* OR “old* man*” OR “old* woman*” OR “old* men*” OR “old* women*” OR “old* LGBT*” OR “old* minorit*” OR senescent OR senile* OR “older adult*” OR “later life*” OR “later in life*” OR “old* vulnerable person*” OR “old vulnerable people*”) N3 (neglect* OR abus* OR maltreat* OR restrain* OR mistreat* OR illtreat* OR “ill treat*” OR ill-treat* OR scorn* OR hit* OR harm* OR damage* OR victimis* OR victimiz* OR trauma* OR manhandl* OR isolate* OR exclu* OR safe-guard* OR safeguard* OR protect* OR exploit* OR bully* OR violen* OR self-neglect* OR intimidat* OR threaten* OR distress* OR abandon* OR fraud* OR rape* OR scam* OR crime* OR cheat* OR extort* OR hoax OR swindle* OR coerce* OR crime* OR criminal* OR assault* OR "bodily harm" OR felony OR batter* OR attack* OR beat*)) OR TI ( ("old* person*" OR “old* people*” OR “old* individual*” OR aged OR gerontol* OR elder* OR geriatric* OR ageing OR aging OR senior* OR retir* OR octogenarian* OR nonagenarian* OR septuagenarian* OR sexagenarian* OR “old* man*” OR “old* woman*” OR “old* men*” OR “old* women*” OR “old* LGBT*” OR “old* minorit*” OR senescent OR senile* OR “older adult*” OR “later life*” OR “later in life*” OR “old* vulnerable person*” OR “old vulnerable people*”) N3 (neglect* OR abus* OR maltreat* OR restrain* OR mistreat* OR illtreat* OR “ill treat*” OR ill-treat* OR scorn* OR hit* OR harm* OR damage* OR victimis* OR victimiz* OR trauma* OR manhandl* OR isolate* OR exclu* OR safe-guard* OR safeguard* OR protect* OR exploit* OR bully* OR violen* OR self-neglect* OR intimidat* OR threaten* OR distress* OR abandon* OR fraud* OR rape* OR scam* OR crime* OR cheat* OR extort* OR hoax OR swindle* OR coerce* OR crime* OR criminal* OR assault* OR "bodily harm" OR felony OR batter* OR attack* OR beat*)) | Expanders - Apply equivalent subjects  Search modes - Boolean/Phrase | Interface - EBSCOhost Research Databases  Search Screen - Advanced Search  Database - MEDLINE | 21,154 |
| S5 | (MH "Elder Abuse") | Expanders - Apply equivalent subjects  Search modes - Boolean/Phrase | Interface - EBSCOhost Research Databases  Search Screen - Advanced Search  Database - MEDLINE | 2,651 |
| S6 | S4 OR S5 | Expanders - Apply equivalent subjects  Search modes - Boolean/Phrase | Interface - EBSCOhost Research Databases  Search Screen - Advanced Search  Database - MEDLINE | 21,964 |
| S7 | S3 AND S6 | Expanders - Apply equivalent subjects  Search modes - Boolean/Phrase | Interface - EBSCOhost Research Databases  Search Screen - Advanced Search  Database - MEDLINE | 751 |

## 4 Appendix 4: Detailed eligibility criteria

##

1. The study must be a systematic review, published either in a peer-reviewed journal or a technical report, according to the following definition:
   - A systematic review is an academic research paper, also called a report, that uses a method called 'evidence synthesis' to look for answers to a pre-defined question.
   - The purpose of a systematic review is to sum up the best available research on that specific question. This is done by synthesizing the results of several studies.
   - A systematic review uses transparent procedures to find, evaluate and synthesize the results of relevant research. Procedures are explicitly defined in advance, to ensure that the exercise is transparent and can be replicated. This practice is also designed to minimize bias.
   - Studies included in a review are screened for quality, so that the findings of a large number of studies can be combined. Peer review is a key part of the process; qualified independent researchers review the author's methods and results.
   - A systematic review must have:
     - Clear inclusion and exclusion criteria
     - An explicit search strategy
     - Systematic coding and analysis of included studies
   - Systematic reviews of quantitative, qualitative and a mixture of quantitative and qualitative studies will be included. In the case of systematic reviews of qualitative studies, they will be included even if they did not appraise the quality of the included primary studies. We will not pre-define types of qualitative study designs of primary studies which we will include. All of the qualitative study designs included in reviews including qualitative studies relevant to prevalence, consequences, risk and protective factors, and interventions for elder abuse will be considered (e.g. participant observation, in-depth interviews, focus groups, etc.).
   - A systematic review may or may not include a meta-analysis. A meta-analysis refers to combining the results of the individual studies in a systematic review to produce an overall statistic.
2. The study must focus exclusively on elder abuse (see definition in Appendix 1) or intimate partner violence against people over 60 years old.
   - Elder abuse refers to a single or repeated act or lack of appropriate action, occurring within any relationship where there is an expectation of trust which causes harm or distress to an older person. The main forms of elder abuse generally recognized and which can occur in the community and institutional settings are physical, psychological, financial/material, sexual abuse and neglect, systemic/organizational abuse, and poly-victimization.
   - Intimate partner violence refers to any behaviour within an intimate relationship that causes physical, psychological or sexual harm to those in the relationship. Examples of types of behaviour include: acts of physical violence, such as slapping, hitting, kicking and beating; sexual violence, including forced sexual intercourse and other forms of sexual coercion, psychological (or emotional) abuse, such as insults, belittling, constant humiliation, intimidation (e.g. destroying things), threats of harm, threats to take away children, and controlling behaviours, including isolating a person from family and friends; monitoring their movements; and restricting access to financial resources, employment, education or medical care.
3. If the study does not focus exclusively on elder abuse or intimate partner violence in people over 60, the relevant findings for elder abuse and intimate partner violence in people 60 years old and over must be disaggregated.
4. If the study focuses on intimate partner violence but includes other age groups, the relevant findings for people 60 years and over must be disaggregated.
5. The study must cover one or more of the following aspects of elder abuse or intimate partner violence in people 60 years and over in the community or in institutions:
   - Prevalence
   - Consequences
   - Risk and protective factors
   - Interventions to prevent, detect, or respond
     - See definitions in Appendix 1
6. If the study covers more than one of these aspects of elder abuse or intimate partner violence in people 60 years and over simulataneoulsy or if it covers one or more of these aspects as well as other aspects of elder abuse or intimate partner violence in people 60 years and over, it will be included.
7. Studies on prevalence must focus on:
   - Population:
     - Older adults (60 or older) who have experienced or are experiencing elder abuse or intimate partner violence;
     - Perpetrators of elder abuse (of any age);
   - Exposure:
     - Exposure to elder abuse or intimate partner violence;
   - Prevalence period:
     - All prevalence periods will be included (past year, since 60, etc.)
8. Studies on risk and protective factors and consequences must focus on:
   - Population:
     - Older adults (60 or older) who have experienced or are experiencing elder abuse or intimate partner violence;
     - Perpetrators (of any age) of elder abuse or intimate partner violence (against a person 60 years or over);
   - Exposure:
     - Exposure to elder abuse or intimate partner violence;
   - Comparator: older adults who have not experienced abuse or intimate partner violence or people of any age who have not perpetrated elder abuse or intimate partner violence.
9. Studies on interventions must focus on:
   - Population:
     - Older adults (60 or older) who are at risk of experiencing, are experiencing, or have experienced elder abuse or intimate partner violence;
     - Perpetrator of elder abuse (of any age);
     - Witnesses of situations of elder abuse (of any age);
     - Non-professional caregivers (family, friends, neighbour, volunteer, etc.) (of any age);
     - Professional caregivers (agency caregivers, nurses, social workers, physicians, etc.) (of any age);
     - General population (awareness campaigns, world abuse awareness day, community level intervention, etc. (of any age));
     - Institutions (care home, long term care facilities, hospitals, banks, etc.);
     - System level (laws and policies changes).
   - Intervention/ exposure:
     - Intervention focused on:
     - Preventing (education, training, awareness campaign, etc.);
     - Detecting (screening guidelines and protocols, detection program, use of detection tools, etc.);
     - Responding, both preventing the recurrence of elder abuse (focusing on perpetrators) and mitigating the impact of elder abuse (focusing on victims). Such programmes may include emotional management training for perpetrators, support for older people, mediation, safeguarding measures, etc.).
   - Outcomes/ consequences:
     - Increase in the detection of elder abuse;
     - Reduction in the occurrence or in the severity of elder abuse;
     - Reduction in risks associated with elder abuse;
     - Increase in reporting;
     - Increase in awareness and general knowledge of elder abuse;
     - Better support for people who are the target of elder abuse and for witnesses or perpetrators.
   - If the study includes a sub-set of eligible interventions, it will be included on condition that separate findings for this sub-set of interventions are available in the study.
10. The study can be in any language. No language restrictions will be applied.
11. The study can have been published at any time. No time restrictions will be applied.

Exclusion criteria

- Systematic reviews of risk factors for health conditions, such as suicide, which could include elder abuse (e.g. elder abuse as a risk factor for suicide) will not be included.
- Protocols of systematic reviews and evidence and gap maps will be excluded from the map, but we will take note of the existence of the protocols to a) check that, if the review or map has already been completed, we have included it; and b) include it in future updates of the map, should the review or map be finished by then.
- Reviews that are not systematic, according to the definition given above.

5 Appendix 5: Screening tool

Figure 5: Screening tool

6 Appendix 6: Critical appraisal tool

AMSTAR-2

https://amstar.ca/docs/AMSTAR-2.pdf

https://amstar.ca/docs/AMSTAR%202-Guidance-document.pdf

https://www.bmj.com/content/bmj/358/bmj.j4008.full.pdf

7 Appendix 7: Advisory Group and Stakeholders Group

**Advisory group**

|  | Name | e-mail | Country | Link/info |
| --- | --- | --- | --- | --- |
| 1. | Yan, Elsie Chau-Wai | elsie.yan@polyu.edu.hk | Hong Kong | https://www.polyu.edu.hk/apss/people/academic-staffs/338-dr-yan-chau-wai-elsie/ |
| 2. | Somers, Susan | sbsomers5@aol.com | USA | President INPEA - http://www.inpea.net/about.html |
| 3. | Kalache, Alexandre | alex.kalache@gmail.com | Brazil | http://ilcbrazil.org/president/ |
| 3. | Rebecca Stoeckle | rstoeckle@edc.org | USA | https://www.edc.org/rebecca-jackson-stoeckle |
| 4. | Isabella Aboderin | isabella.aboderin@  bristol.ac.uk | UK/Africa | http://www.bristol.ac.uk/sps/people/isabella-a-aboderin/index.html |
| 5. | Mark Bellis | m.a.bellis@bangor.ac.uk | UK | https://phw.nhs.wales/about-us/board-and-executive-team/board-members/mark-bellis/ |
| 7 | Maria Isolina Dabove | isolinadabove@gmail.com | Argentina | https://salud.gob.ar/dels/autores/dabove-maria-isolina |

**Stakeholders’ group**

|  | Name | e-mail | Country | Link or info |
| --- | --- | --- | --- | --- |
| 1. | Hussein, Shereen | S.A.Hussein@kent.ac.uk | Egypt-UK | https://www.menarah.org/author/shereen/ |
| 2. | Goergen, Thomas | thomas.goergen@dhpol.de | Germany | https://www.dhpol.de/departements/departement_III/FG_III.1/goergen.php  https://www.researchgate.net/profile/Thomas_Goergen |
| 3. | Nhongo, Tavenga | nhongo@wananchi.com | Kenya | INPEA Director - https://bit.ly/3lULQhB  http://www.inpea.net/reports-resources/archives2011/ |
| 4. | Barratt, Jane | jbarratt@ifa.ngo | Canada | https://ifa.ngo/team/secretariat/dr-jane-barratt/ |
| 5. | Sleap, Bridget | bsleap@helpage.org | UK | https://www.linkedin.com/in/bridgetsleap/?originalSubdomain=uk |
| 6. | Hada, Rio | rhada@ohchr.org | Switzerland | Office of the United Nations High Commissioner for Human Rights (OHCHR) |
| 7. | Kiran Pradhan, Prabhat | prabhatkiranpradhan@gmail.com | Nepal | https://www.linkedin.com/in/prabhat-kiran-pradhan-14936b6b/?originalSubdomain=np |
| 8. | Emem Omokaro | emem@daveomokarofoundation.org | Nigeria | https://www.thedaveomokarofoundation.org/ |
| 9. | Hamby, Sherry | sherry.hamby@sewanee.edu. | USA | https://new.sewanee.edu/programs-of-study/psychology/faculty-staff/sherry-l-hamby/ |
| 10. | Arrue, Borja | Borja.Arrue@age-platform.eu | EU | Https://www.age-platform.eu/ |
| 12. | Shankardass, Mala | mkshankardass@gmail.com | India | https://www.researchgate.net/profile/Mala_Shankardass  INPEA Regional Rep Asia |
| 13. | Laura Tamblyn Watts | ltamblynwatts@gmail.com | Canada | https://www.canage.ca/team |
| 14. | Ariela Lowenstein | ariela@research.haifa.ac.il | Israel | https://expertfile.com/experts/profariela.lowenstein/prof-ariela-lowenstein |
| 15. | Kate Wilber | wilber@usc.edu | USA | https://gero.usc.edu/faculty/wilber/ |
| 16. | Olayinka Ajomale | info@cadrop.org | Nigeria | Centre on Ageing, Development and Rights of Older Persons www.cadrop.org |
| 17. | Yongming Liu | cardtonm@263.net | China | Director of the Clinical Research Center of geriatric diseases in Gansu Province  https://www.lzdxdyyy.com/zjdetail.aspx?ID=113 |
| 18. | Ying Wang | wangyingever@lzu.edu.cn | China | Senior Researcher of the "Aging Society and Education for the Aged" of the All-China Women's Federation (ACWF)  http://www.women.org.cn/art/2013/7/10/art_213_103328.html |
| 19. | Tracy Howe | proftraceyhowe@gmail.com | UK | Cochrane-Campbell Global Ageing/University of Manchester |
| 20. | Silvia Perel-Levin | s.perel58@gmail.com | Switzerland | NGO Committee on Ageing |
| 21. | Georgina Veitch | georgina.veitch@helpage.org | UK | HelpAge International |
| 22. | Claire Wan Yuen Choo | ccwy@um.edu.my | Malaysia | University of Malaya |

**Report on 17 January 2021 Stakeholders Meeting on evidence and gap map and mega-map on elder abuse**

**Background**

It is widely recognized that the global priority of elder abuse is not commensurate with the burden of the problem globally and that major gaps remain in data and research on all aspect of the topic.

In the past 20 years, WHO has addressed elder abuse, but somewhat sporadically. It has not given it the same attention as other forms of violence such as violence against women and violence against children. With the UN’s declaration of the UN Decade of Healthy Ageing 2021-2030, WHO has decided to increase its activities in the area of elder abuse. As a first step it is conducting two projects which will inform the development of an evidence-based strategy to address elder.

The first project is exploring the factors that account for the inadequate political priority of elder abuse with a view to devising a strategy to addressing these. The second project, the subject of this Stakeholders Meeting, aims to map evidence on all aspects of elder abuse – its prevalence and consequences, risk and protective factors for elder abuse, and interventions to prevent, detect, and respond to elder abuse.

An evidence and gap map displays the available evidence relevant to a specific topic or research question based on primary studies. It provides a visual and interactive display of the existing evidence, which is updated on a regular basis. A typical map is a matrix of intervention categories (rows) and outcome domains (columns), with filters which allow the evidence to be grouped in different ways (e.g. by study design, by region, or country). See here for a recent example of an evidence and gap map on interventions for reducing violence against children in low- and middle-income countries.

A “megamap” is similar in many respects to an evidence and gap map but is generally broader in scope covering a larger sector and is based only on systematic reviews (and other maps if they exist) and not on primary studies. An example of a megamap on child wellbeing, produced by the Campbell Collaboration Secretariat for UNICEF, can be found here.

Such maps serve several critical purposes in any evidence-based approach to addressing a health or social problem. For instance, they increase the discoverability, accessibility, and use of the evidence on a particular topic. They identify gaps in the evidence that should be prioritized in research agendas. They guide implementers towards interventions that work and away from interventions that are waste of money. They are a necessary first step in producing higher-level evidence products for policy- and decision-makers, such as evidence platforms and portals, evidence-based guidelines, and evidence-based policy briefs (see here for more discussion of this approach).

Given the particularly scarce resources available for elder abuse globally and the slow progress made to date in developing effective interventions, it is an ethical imperative to adopt a rigorously evidence-based approach, and evidence and gap maps play a central role in any such approach. An evidence-based approach to elder abuse can maximize the impact of policies and interventions to address elder abuse and reduce the actual number of older people being abused and neglected in a measurable way.

**Objectives**

The purpose of this online workshop was twofold:

To familiarize key stakeholders with the nature and purposes of evidence and gap maps and mega-maps;

To consult key stakeholders on the scope and framework for:

The mega-map (i.e. based on systematic reviews) that WHO and the University of Sherbrooke in Canada are proposing to do on the prevalence and consequences of, and risk and protective factors for, elder abuse, as well on interventions to prevent, detect, and respond to elder abuse;

The evidence and gap map (i.e. based on primary studies and systematic reviews) that Lanzhou University in China is proposing to carry out on interventions to prevent, detect, and respond to elder abuse.

**Participants**

The approximately 35 Stakeholders and Members of the Advisory Group who participated in this meeting were selected based on their expertise in different areas of elder abuse and with a view to ensuring balanced geographical and gender representation.

Meeting programme

This online meeting lasted over three hours, from 1pm to after 4.25pm CET.

1.00: Welcome & the role of evidence maps in building the evidence architecture by Howard White, Campbell Collaboration, followed by Q&A

1.30: Plans for the use WHO elder abuse evidence maps by Chris Mikton, WHO

1.45: Presentation on the elder abuse evidence and map on prevention, detection, and response interventions by Jieyun Li, Lanzhou University, followed by a framework consultation exercise in small groups introduced by Howard White, CEO Campbell Collaboration

2.30: Short break

2.40: Feedback from groups

3.00: Presentation on the elder megamap on the prevalence and consequences of, and risk and protective factors and interventions for, elder abuse scope by Yongjie Yon, WHO, and Marie Beaulieu, University of Sherbrooke followed by a framework consultation exercise in small groups introduced by Howard White, Campbell

3.40: Feedback from groups

3.55: Wrap up and close.

Evidence and gap maps: their nature and uses

There were two presentations on the nature and uses of evidence and gap maps. The first, by Howard White, defined evidence and gap maps, described their main uses, and explained the key role they play in the building the “evidence architecture” for any field. Such maps are a prerequisite for developing evidence-based decision-making products for policy- and decision-makers, such as evidence portals, guidelines, and checklists. Evidence and gap maps tell us about the current state of data, studies, systematic reviews, and databases on a particular subject, on which the evidence-based decision-making products are based.

The second presentation described the somewhat sporadic history of WHO’s efforts to address elder abuse and its current plans to step up its activities in this area. It emphasized the direct relevance of evidence and gap maps to most of WHO’s core functions (e.g. shaping the health research agenda, setting norms and standards via guidelines, articulating evidence-based policy options) and their importance in informing the strategy WHO is developing to address elder abuse. WHO is planning to use these maps to identify research gaps, commission research to fill these gaps in a more coordinated and strategic way, to produce higher-level evidence products for decision-makers (e.g. guidelines), to produce policy briefs, and to guide programme implementers in countries.

The evidence and gap map on elder abuse interventions (Lanzhou University)

**Presentation**

Jieyun Li from Lanzhou University in China gave a presentation on the evidence and gap map they are planning to carry out on interventions to detect, prevent, and respond to elder abuse. The overarching question she addressed was how best to organize the large number of primary studies evaluating the effectiveness of elder abuse interventions. What is the optimal classification of interventions and outcomes – i.e., overall framework (see Annex 1). She presented the proposed classification of interventions, the proposed classification of outcomes measured by the studies, as well as the different filters by which studies might be grouped (e.g. type of abuse, region, quality of study).

**Consultation**

Participants, who broke up into smaller groups, were asked to discuss the framework that Lanzhou University is proposing to use, code a few studies using the framework to test the framework, and discuss any other issues relevant to the proposed evidence and gap map on interventions for elder abuse. The main issues discussed were as follows:

Opinion was divided on whether the standard definition of elder abuse (https://www.who.int/ageing/projects/elder_abuse/en) needed to be revised. Some participants maintained we needed to replace it by “a 21^st^ century definition of elder abuse”. Others cautioned against re-opening the debate on definitions and noted that the consensus supporting this definition was hard-won and was an important factor in the scientific progress that had been made in the field in the last decades. Still others suggested the standard definition be retained as a core definition, but additional dimensions be added. Some of the main problems with the definition raised were as follows: expectation of trust should be reconsidered; inclusion of abuse – particularly scams – by strangers and technological-based abuse such as online or telephone abuse; cross-cultural relevance of the definition and its ability to capture practices such as witchcraft accusations, abandonment; the overlap with ageism; whether self-neglect should be included; and the need to capture systemic abuse better. Several people praised the definition of the New York Center for Elder Abuse – https://nyceac.org/about/definition/;

The need to include grey literature (including books, book chapters, and reports) which is more likely to cover elder abuse occurring in low- and middle-income countries;

The gender and intersectional perspective should be more prominent in the framework;

Doubts that the intervention categories were sufficiently granular were raised;

The need to add a separate category for “psychosocial interventions” (which are currently included under educational), policies, jurisprudence, and legislation;

That the outcome categories were too vague and need to be specified in greater detail;

The mega-map on the prevalence and consequences of and risk and protective factors and interventions for elder abuse (WHO, University of Sherbrooke)

**Presentation**

Yongjie Yon of the WHO European Office and Marie Beaulieu of Sherbrooke University gave a presentation on the mega-map on prevalence, consequences, risk and protective factors, and interventions. The main concern was once again how best to organize the information in the many systematic reviews that will be included in the mega-map in terms of the rows and columns (i.e. the framework, see Annex 2), as well as the filters, for each of the maps on prevalence, consequences, risk and protective factors, and interventions.

**Consultation**

Participants, who again broke up into groups, were asked to discuss which kinds of reviews should be eligible for the megamap (e.g. whether qualitative reviews should also be included) and to consider a few reviews as examples, whether there were topic areas that we missing, the adequacy of the framework that was proposed for the megamap, and to consider any other issues relevant to the proposed megamap. The main issues discussed in this second consultation were as follows:

- That the outcome categories, which include the main different types of abuse, should also include the co-occurrence of multiple types of abuse and poly-victimization;
- The framework for consequences should allow for more than one type of consequence to be selected;
- Social and economic conditions need to be explicitly included, particularly among the consequences of elder abuse;
- Consequences for social service use (e.g. APS, supportive shelter) should be more explicitly included in the framework for the map on consequences;
- The distinction between community and institution is not fine-grained enough and fails to capture formal and informal caregiving arrangements in the community;
- Regarding the outcomes – types of abuse – some measure of severity should be considered;
- Among interventions, jurisprudence, laws and policies should be included as a separate type of interventions;
- We should consider including qualitative reviews, particularly for consequences and perhaps for interventions too;
- Publication bias should be considered;
- The domain of help-seeking behaviors should be included.

Comments in the chat function

- Some of the main feedback provided via the chat function in zoom were as follows:
- That evidence synthesis processes, such as these maps, are slow and fail to capture important recent development like the changing nature and increasing rates of elder abuse during the COVID-19 pandemic.
- Concerns were raised about the inclusion of older people themselves in the consultation process and more details about this process after this meeting were requested.
- Several people asked for systemic abuse and systemic factors that facilitate abuse, jurisprudence and legislation (which are distinct), online/digital scams and frauds and online/digital psychological and sexual abuse, and self-neglect and its link to systemic abuse to be more clearly included.
- One of the biggest accomplishments in the field of elder abuse in recent decades was the development of a definition of elder abuse on which there is some scientific consensus. Broadening the definition to include other negative outcomes and actions towards older people – such as crimes by strangers, ageism, or self-neglect – would be problematic and damaging to science and roll back the significant progress that has been made over the years.
- Add costs to the map on consequences.
- It is important that we include grey literature and literature that is harder to access. This will be important to ensure that elder abuse in low- and middle-income countries is covered, as some of this literature is not published in peer-reviewed publications and cannot be accessed through the usual electronic databases, including those for grey literature. Ensuring this literature is included might, it was pointed out, require engaging in some detective work to identify it. This might involve, for example, searching organizations’ websites, searching for literature in multiple languages, and talking with key informants from the regions and organizations in question.

**Next steps**

The Lanzhou team and the WHO/Sherbrooke team will revise their respective frameworks in light of the feedback received by meeting participants.

By April 2021, each team will develop a protocol, which will be circulated to participants for comments.

By the end of November, first draft of the maps will be circulated to stakeholders for review.

Appendix 5: Screening tool
